# Active Infrared Thermography for Polymer Matrix Composites: Heat-Generation Mechanisms, Vibrothermography, and Signal Processing

**DOI:** 10.3390/ma19214742

**Published:** 2026-11-08

**Authors:** Mohammed Abdulkedir Alfeki, Andrzej Katunin

**Affiliations:** Department of Fundamentals of Machinery Design, Faculty of Mechanical Engineering, Silesian University of Technology, Konarskiego 18A, 44-100 Gliwice, Poland

**Keywords:** active infrared thermography, polymer matrix composites, vibrothermography, local defect resonance, self-heating, non-destructive testing, structural health monitoring

## Abstract

Active infrared thermography operates without contact, covers the full field from a single side, and can operate at a limited temperature rise, which makes it well suited to the inspection of polymer–matrix composites. However, its diagnostic performance is governed less by the detector hardware than by the mechanism through which heat is generated at, or transported to, damage. This review compares optically and mechanically excited thermography from that standpoint. The underlying material physics is summarized first: diffusive transport and the loss of contrast with damage depth for externally heated configurations, coupled thermoviscoelastic dissipation for internally heated ones, and the glass-transition constraint that limits admissible excitation power in polymers. Pulsed, lock-in, step-heating, pulsed-phase, and frequency-modulated modalities are then reviewed with the depth inversion relations they support. Mechanical excitation is treated in three branches: friction dominated ultrasonic thermography, local defect resonance as an excitation strategy, and self-heating based vibrothermography (SHVT) as a viscoelastic heat-generation mechanism. The reported detection capability of SHVT is then analyzed. Two limits emerge: one set by the size of the damage, the other by its position through the thickness. Finding damage and measuring its size also prove to be different capabilities with different limits. Signal processing is reviewed in three groups, namely classical sequence-based transforms, procedures for background suppression and quantitative characterization, and machine learning-based methods. Open issues in quantification, benchmarking, and data availability are noted, along with the limited use of machine learning-based vibrothermography processing.

## 1. Introduction

**Figure 1:**
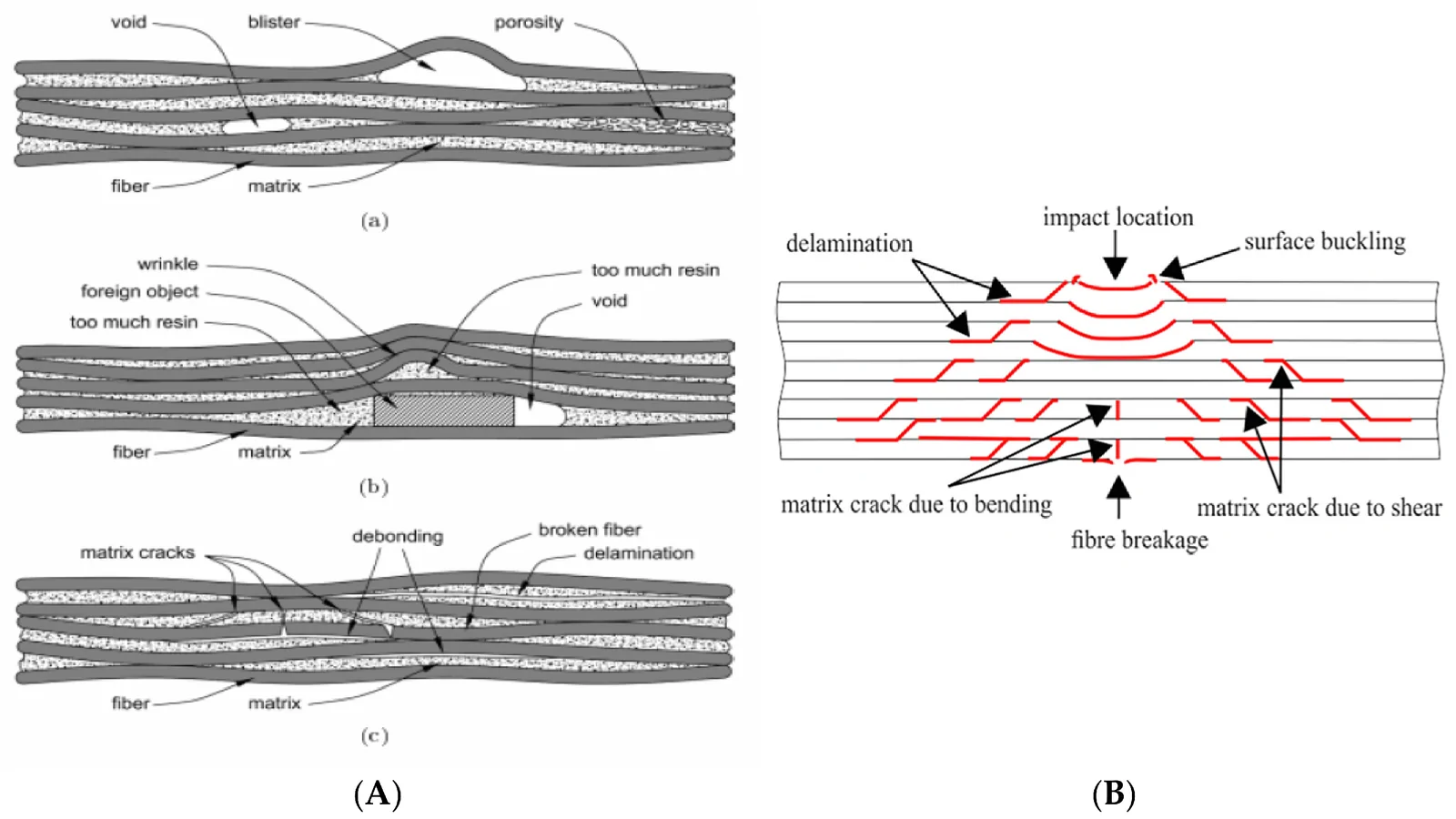
Typical flaws in PMCs: (**A**) defects occurring during the manufacturing process and (**B**) impact damage in fiber-reinforced polymer composite structures [4]. The three panels of (**A**), labelled (a–c) in the reproduced original, show voids, a blister and porosity; a wrinkle, a foreign object and excess resin; and matrix cracks, debonding, broken fibers and delamination, respectively.

The main concern with polymer matrix composites (PMCs) is flaws, understood as defects and damage that can be difficult to detect yet strongly influence performance. Despite good characteristics, PMCs are very sensitive to manufacturing defects and service-induced damage, like voids or porosity, waviness of the fibers, cracks in the matrix, poor fiber-matrix bonding, barely visible impact damage (BVID), and delamination between layers, as shown in Figure 1, all of which cause a significant reduction in strength and performance [1,2]. They also act as stress risers because once the crack starts, the load transfer between fiber and matrix becomes inefficient. Structural flaws vary based on their size, nature, morphology, shape, and severity. In practice, flaws can be surface-visible, near-surface, or fully internal, with subsurface scenarios posing particular risk due to limited detectability using visual inspection alone. A particularly challenging in-service scenario is impact damage that can be non-obvious on the surface but severe internally, often manifesting as interlaminar cracks with associated matrix cracking, like BVID. Understanding flaw formation in PMCs is essential for structural integrity and reliability. Due to this, lifecycle assurance, structural health monitoring, maintenance planning, and certification procedures need effective, efficient, and non-destructive inspection techniques to detect and quantify such flaws on time [3].

A wide set of alternative non-destructive testing (NDT) techniques are used for composites, such as visual inspection, tap testing, penetrant testing, radiography, shearography, ultrasonic testing, infrared thermography (IRT), X-ray computed tomography (CT), and others [5]. Each technique presents distinct trade-offs in terms of coverage area, depth sensitivity, damage detectability, access requirements, operational cost, and inspection rate [3]. Among these NDT techniques, active IRT has gained significant prominence due to its non-contact nature, full-field inspection capability, and rapid damage exploration in subsurface regions of composite structures [6,7]. A problem that still requires a solution for PMCs today is improving thermography’s reliability and quantification under realistic constraints, including the need to limit excitation power to avoid overheating or material changes and the need to detect subtle, near-surface-to-subsurface flaws with high contrast.

In recent years, the application of IRT in NDT has been increasing due to its mentioned unique properties. Although there are different effective NDT techniques applicable to PMCs, as listed above, IRT is one of the most suitable NDT techniques for PMC structures, which finds confirmation in considering this class of techniques in the list of NDT techniques approved by the Federal Aviation Administration for aircraft inspections [7,8,9,10]. An approval sets the conditions under which a technique is accepted; it does not state a general capability. In an airframe inspection, access is often restricted to one side, the surface carries paint, sealant or a lightning-protection mesh of differing emissivity, ambient conditions are uncontrolled, and curvature, stiffeners and fastener rows distort the one-dimensional heat flow assumed in Section 3. Inspection time is set by the maintenance schedule. These constraints explain why laboratory detection limits do not transfer directly to in-service practice.

When using IRT, characterizing a flaw requires uniform heating, high contrast, and good control of background noise. However, obtaining such behavior is difficult in a PMC structure due to emissivity variation, composite architecture, dependency on excitation parameters or nature of loading, thermal and mechanical boundary conditions, and anisotropic properties [7].

In the case of PMCs, it is necessary to understand their thermal, mechanical, and viscoelastic nature to get the minimum required temperature distribution over the surface that is enough to detect a flaw at any location, where the flaw disrupts heat flow and produces measurable thermal contrast. Numerous mechanisms and principles exist by which heat is applied to a tested structure in IRT, differing mainly in the source (internal or external), the heating function or waveform, and the underlying principle. The various excitation modalities of active IRT include optical (flash/lamp and laser), electromagnetic (induction, especially in conductive carbon fiber systems), microwave, and mechanical (vibrothermography) modalities. These can be further divided according to heating functionality or waveforms as pulsed, lock-in, pulsed phase [11], step heating [7], frequency modulated, and long pulse thermography (LPT) [3,12]. Thermal contrast, irrespective of modality or heating operation, is affected by the process of heat generation at damage locations, thermal diffusion in the composite material, and the variations in temperature on the surface. Therefore, thermal control is needed to keep the surface temperature below glass-transition temperatures ($T_{g}$), which might introduce irreversible changes in the tested structures [13].

Two emerging complementary mechanical excitation strategies have already shown specific promises with PMC inspection: local defect resonance (LDR) and self-heating based vibrothermography (SHVT). LDR makes use of defect-related resonant frequencies to realize efficient heating at low power levels [14,15]. Excitation frequency and the shape of the damage are also vital to the performance of LDR [16,17,18,19,20]. However, SHVT takes advantage of natural frequencies as well as multiple resonances, which lead to viscoelastic heat dissipation to generate distributed heating with extensive coverage, often with a higher temperature tolerance and effective flaw measurement (e.g., matrix cracking, BVID) at lower power levels [15].

In light of the mentioned mechanical excitation strategies, the unique heat generation approach should be considered. Polymers have a viscoelastic nature that distinguishes them from other materials. This viscoelastic nature allows polymers to dissipate heat under cyclic loading; it is also one of the constraints on damage inspection, which is why an understanding of the coupled thermoviscoelastic behavior is important. Several studies have addressed the modeling of the thermoviscoelasticity of polymers with different geometrical properties [21,22,23]. Another constraint is the thermal nature of polymer-based structures, since polymers are characterized by a low $T_{g}$; an internal temperature increasing towards $T_{g}$ can have catastrophic consequences [23]. The material properties place strict limits on the admissible temperature levels: if the temperature unexpectedly surpasses these thermal safety limits, irreversible changes or local overheating are introduced, especially in low-$T_{g}$ thermosets and thermoplastics. This led to the development of IRT techniques that provide sufficient thermal contrast at the damage with lower power consumption and minimum temperature change, exploiting the structural dynamics of the inspected composite material.

Although there have been significant improvements in IRT hardware and dynamic modalities, which have enhanced the detectability of subsurface damage, there is still limited comparative knowledge of how the heat generation mechanisms and practical constraints dictate performance in PMCs, particularly in optical, LDR, and SHVT modalities. More recent technological innovations in detector technology, such as mercury cadmium telluride, indium antimonide, quantum well infrared photodetectors, and new quantum-dot devices, with the aid of smart sensor integration and multispectral or terahertz-assisted imaging, have further extended diagnostic capability [24].

Although research on fundamental thermographic modalities has been conducted in the last twenty years, there is limited literature on the comparison of heat generation processes, detection limits, and feasibility constraints of optical and vibration-based methods applied to PMCs. Moreover, some reviews have covered IRT in general or optical methods in detail [3], while the link between the viscoelastic characteristics of the material, the shape of the damage, and the optimum excitation parameters is limited. In relation to the review done by [3,12], this review is organized instead by the physical mechanism through which heat reaches the damage, and it treats viscoelastic hysteretic heating, which neither survey covers in depth, as a heat-generation mechanism in its own right. Three things follow. Heat generation is linked to the thermoviscoelastic behaviour of the matrix and to the geometry of the damage rather than to the equipment. The performance of each modality is given together with the conditions that produced it. And the gaps that remain in standardization, benchmarking and data availability are identified.

## 2. Introductory Fundamentals of Thermography

In the assessment of structural integrity, the role of NDT is essential, since some structures are very sensitive to flaws, and their performance is influenced by these flaws. Therefore, NDT is the most essential tool for assessing structural conditions. Before addressing the specific NDT techniques, a general comparison is summarized in Table 1.

As the manufacturing sophistication, complexity, and usage of PMCs increases, there is a proportional increase in the need for maintenance and inspection. This advancement in composite materials has encouraged the development of many inspection approaches or NDT techniques.

The comparison and selection of NDT techniques depend on several factors presented in Table 1: flaw type, inspection scenario, minimum detectable flaw size, allowable damage tolerance, and inspection interval, together with reliability, repeatability, ease of operation, and applicability outside laboratory conditions. Cost-effectiveness is generally the decisive factor in practice, and accessibility is a further constraint where components cannot readily be disassembled. In many cases, a single method does not provide sufficient information about material integrity, so a suitable combination of techniques is required [25].

BVID presents a particular challenge, since surface indications are minimal while significant subsurface delamination or matrix cracking exists [8]. Consequently, IRT is frequently combined with complementary NDT methods to fully characterize BVID in polymer-based composites. Ultrasonic testing (conventional C-scan or phased array) remains the most widely used reference technique, providing higher through-thickness resolution and more reliable depth sizing of delaminations than IRT alone, though at the cost of longer inspection times and the need for coupling [26]. X-ray CT offers the highest spatial resolution and can resolve complex three-dimensional damage morphology, making it valuable as a ground-truth reference against which faster field techniques can be validated, albeit at considerably higher cost and with limited applicability outside the laboratory [27]. Eddy current testing complements IRT for near-surface damage in conductive or hybrid composites, while laser Doppler vibrometry (LDV) is increasingly used to identify the vibrational signatures of BVID independently of thermal measurement, which is particularly useful for locating the LDR frequencies exploited in vibrothermography (Section 4.2) [14]. In practice, full characterization of BVID is quite challenging, and combining fast, full-field IRT screening with a higher-resolution confirmatory technique remains the most reliable inspection strategy [8,26].

### 2.1. Principles of Infrared Thermography

IRT is one of the NDT techniques; its principle of operation is based on capturing the thermal radiation of a material under test (MUT) at a temperature above absolute zero using a thermal (infrared) camera. It operates according to the blackbody radiation law developed by Planck [28,29].

IRT operates within the infrared portion of the electromagnetic spectrum, conventionally taken to extend from approximately 0.75 to 1000 micrometers. Because infrared radiation from opaque solids originates within a very thin surface layer, thermography measures a surface temperature rather than a bulk temperature. In NDT, the emitted radiation is detected principally in the mid-wave infrared (MWIR) and long-wave infrared (LWIR) regions, which lie outside the range of human vision.

Electromagnetic radiation is conventionally partitioned into wavelength “bands,” and within this scheme the infrared portion of the spectrum is commonly split into four ranges, with boundaries that are conventional rather than physically sharp: near-infrared (0.75–3 µm), MWIR (3–6 µm), LWIR (6–15 µm), and extreme infrared (15–1000 µm) [30,31]. NDT practice concentrates almost exclusively on the MWIR and LWIR ranges, a preference driven by two practical factors: these bands offer strong thermal contrast across most engineering materials, and they coincide with windows of good atmospheric transmission [32,33,34]. Differentiating Planck’s radiation law with respect to wavelength and solving for the peak intensity yields Wien’s displacement law, Equation (1) [31]

(1) $$\lambda_{max}=\frac{2898}{T}.$$

This relationship reflects the common experience that the color of an object that is heated is observed to change from red to orange and yellow as the temperature T rises.

An IRT system pairs a camera having interchangeable optics with software for processing the resulting thermograms. Inside the camera, there is the infrared detector, which converts incident infrared radiation emitted by the target surface into an electrical signal (voltage or current). While emitted energy scales with surface temperature for any object, what the detector actually records is modulated by the emissivity of a surface [31].

These raw detector readings are then used to obtain the apparent temperature values using a technique called radiometric calibration that takes into consideration the characteristics of the detector and the emissivity of the surface being inspected, which varies as a function of wavelength.

### 2.2. Material Properties and Thermal Response

Damage visibility in composite inspection is governed jointly by material characteristics, namely thermal (conductivity, diffusivity, effusivity, specific heat), spectral (emissivity, absorptivity, reflectivity, transmissivity), and physical (density, porosity) [34], and by external factors such as ambient conditions and detector sensitivity, both of which shape the achievable thermal contrast.

Heat propagation through a PMC or other MUT follows the Fourier diffusion equation [31,35]:

(2) $$\frac{\partial T}{\partial t}=\alpha \nabla^{2}T,$$

where t denotes time and α is the material’s thermal diffusivity. For an isotropic medium, α is given by Equation (3), with k, which is the thermal conductivity, ρ is density, and $c_{p}$ is specific heat capacity [36].

(3) $$\alpha =\frac{k}{\rho c_{p}}.$$

A relative thermal diffusivity $\alpha_{r}$ is defined as the difference between the diffusivity of the intact material $\alpha_{s}$ and that of the damaged region $\alpha_{d}$, so that $\alpha_{r}=\alpha_{s}-\alpha_{d}$. Two regimes follow: when $\alpha_{s}>\alpha_{d}$ the relative diffusivity is positive, and when $\alpha_{s}<\alpha_{d}$ it is negative. This sign carries through to the thermal contrast $C_{T}$, i.e., the difference in surface temperature between the damaged and the sound regions during transient heating, so that $C_{T}$ is positive when $\alpha_{r}$ is positive and negative when $\alpha_{r}$ is negative.

As stated above, the visibility of damage depends on several factors, which include material characteristics, atmospheric conditions, and instrument sensitivity. $C_{T}$ is enhanced with increasing $\alpha_{r}$, but it is affected by both the size and depth of the damage; more specifically, $C_{T}$ is low for small, damaged areas and weakens as the depth z increases. According to the analysis reported in [31], $C_{T}$ is assumed to be inversely proportional to the cube of the damage depth (z), as expressed in Equation (4):

(4) $$C_{T}\propto \frac{1}{z^{3}}.$$

Flaws interrupt natural thermal conductivity paths because they have a different thermal conductivity than the sound material or healthy region [37,38,39]. These discontinuities become thermal resistance, preventing the diffusion of heat and creating local temperature changes. When using reflection modes or active heating strategies, damaged regions normally appear warmer, and when using cooling or transmission mode strategies, damaged regions appear cooler. These temperature variations are the basis on which damage is detected in IRT.

Despite this qualitative understanding, a fully validated quantitative relationship linking damage characteristics (size, shape, orientation, and interface condition), the mechanical or thermal energy dissipated at the damage, and the resulting surface thermal field has not yet been established. For optically excited IRT, the surface temperature field is governed by linear diffusive heat transport from an external source, for which the underlying physics, described by Equations (2)–(4), is comparatively well characterized. However, once heat is generated internally at the damage itself, as occurs in mechanically or vibration-excited thermography, the coupling between damage geometry, contact and interface physics (friction, clapping, hysteretic damping), and the resulting local heat flux becomes strongly nonlinear and case-dependent [40,41,42]. Existing models capture individual dissipation mechanisms in isolation, typically friction-dominated heating at open or partially closed cracks and viscoelastic hysteretic heating in polymer matrices, but a general, experimentally validated relationship applicable across damage types, geometries, and materials remains an open research problem, which is discussed further in the context of vibrothermographic techniques in Section 4.

### 2.3. Classification of Infrared Thermography Techniques

**Figure 2:**
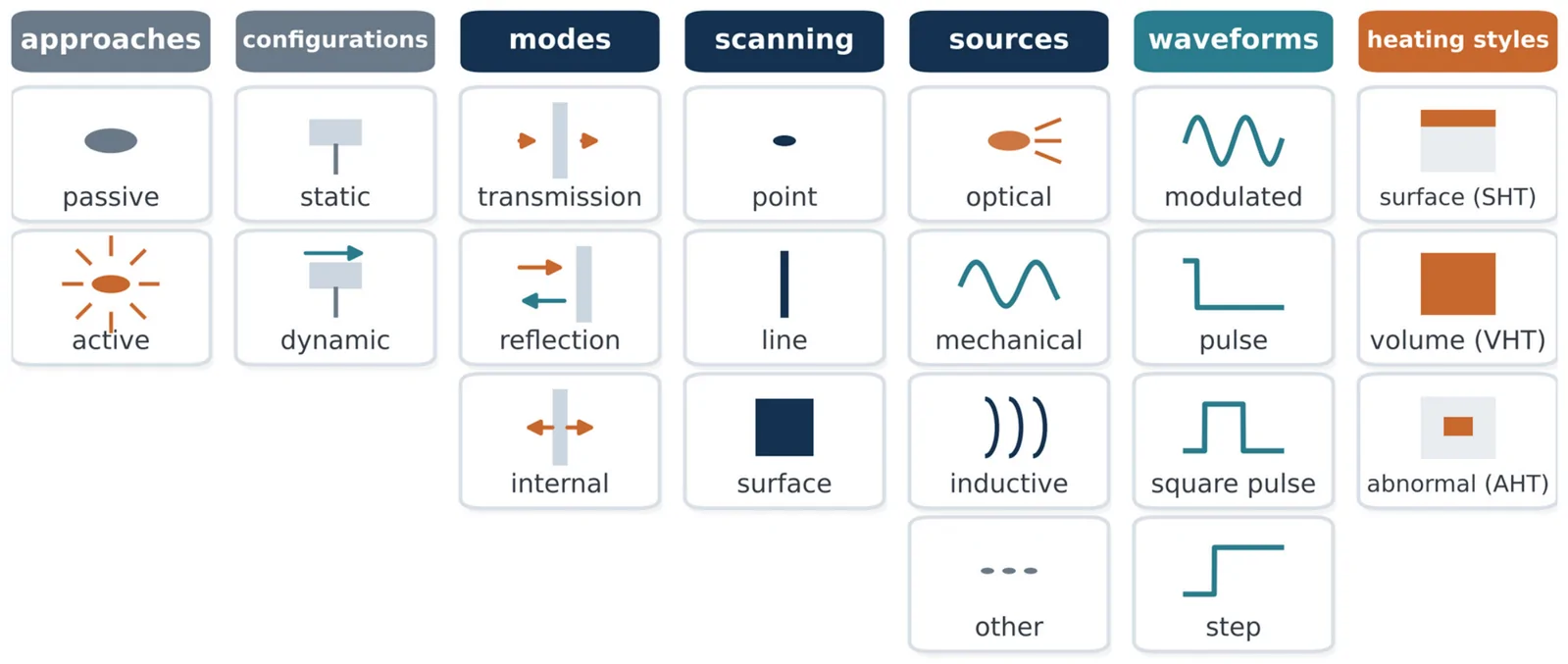
Elements to consider in a thermography inspection scenario and classification of IRT. Diagram drawn with the assistance of Claude (Anthropic, claude.ai); the content was specified and verified by the authors.

IRT testing techniques can generally be categorized as either passive or active approaches, depending on the need for sources of energy, as indicated in Figure 2 [42]. Passive thermography makes use of natural thermal situations without external stimulation, and it is used in health monitoring of structures. Although noninvasive and convenient, passive methods usually have low spatial resolution and cannot be used well to detect deep-seated damage.

Active IRT, however, also utilizes sources of energy (optical, mechanical, electromagnetic, or electrical) to produce adjusted thermal gradients as shown in Figure 2 [12]. Optical sources include cinematographic lamps, quartz line infrared lamps, photographic flash sources, and laser beams [8]; mechanical sources include vibration and ultrasonic/sound wave excitation, while electrical sources include eddy current excitation, electromagnetic waves, and direct electrical current heating [43,44].

Figure 2 indicates that IRT taxonomy depends on various key elements. These key elements are the most important aspects, which are the energy sources, configuration, modes of heating, scanning mechanism, sources of energy used, excitation forms, and heating style.

**Figure 3:**
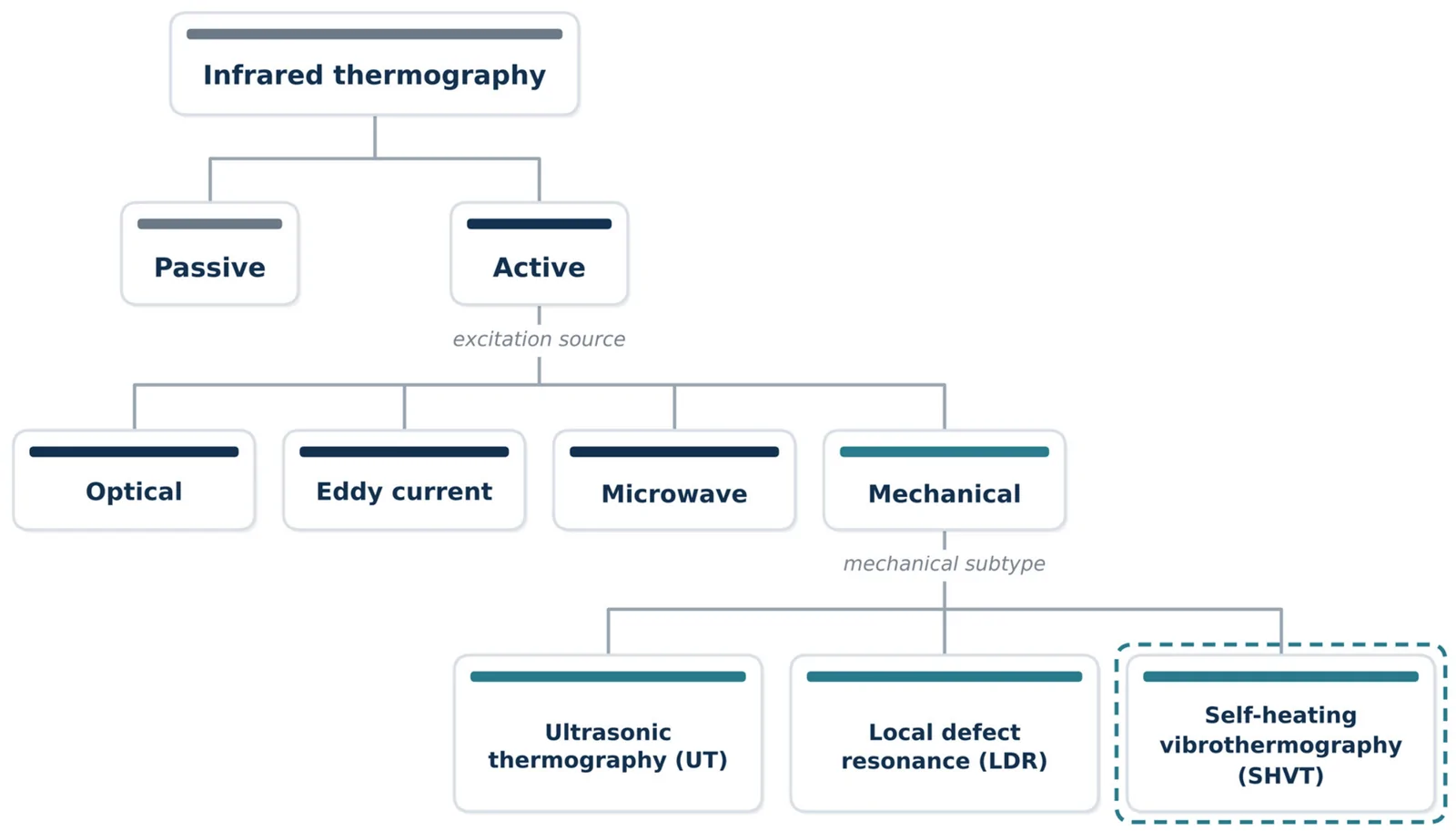
Classification of IRT for PMC NDT based on excitation sources. Diagram drawn with the assistance of Claude (Anthropic, claude.ai); the content was specified and verified by the authors.

As shown in Figure 3, this classification forms a hierarchy: at the top level, IRT is divided into passive IRT and active IRT, depending on whether external stimulation is used. Active IRT is further subdivided by the type of energy source into optical, eddy current, microwave, and mechanical (vibrothermographic) excitation. The mechanical branch itself splits into ultrasonic thermography, which relies primarily on frictional heat generation at crack faces under high-frequency excitation; LDR, which tunes the excitation frequency to a resonance localized at the damage to concentrate vibrational energy there; and SHVT, which exploits the intrinsic viscoelastic hysteresis of the material itself as the heat source. These three mechanical branches are discussed in detail in Section 4, while the optical branch is the subject of Section 3.

The other ways of classifying IRT are based on the methods and principles of excitation shown in Figure 3. However, detailed analysis of these techniques is necessary regarding the behavior of the polymer and the excitation strategy.

Vibration-based (mechanical) thermographic NDT is governed by a distinct set of operating parameters compared with optical excitation. The principal parameters include the excitation frequency, the excitation amplitude or input power, the coupling method (contact transducers, horns, or shakers versus non-contact acoustic excitation), boundary conditions, and the excitation duration. A central constraint of resonance-based approaches, such as LDR (see Section 4.2), is the need for prior knowledge of the defect’s resonance frequency, usually obtained from an auxiliary vibrometry measurement, which adds complexity relative to a single-step thermal scan [45,46,47]. Excitation power is likewise constrained by the requirement to remain genuinely non-destructive, limiting the achievable thermal contrast, particularly for weakly dissipative damage. The primary goals of vibration-based thermography are therefore to achieve reproducible, quantifiable thermal contrast using the lowest input power that doesn’t cause structural damage, to reduce dependence on a priori knowledge of damage location or resonance frequency, and to extend applicability for which optical excitation provides limited depth penetration or contrast [12,48].

From the operational point of view, IRT is either static, where the camera, excitation source, and object remain fixed, or dynamic, where one of these elements moves during inspection, as indicated in Figure 2 under configuration.

In active IRT, energy can be introduced or generated in different ways. The heat transfer modes are transmission, reflection/external excitation, or internal excitation, as indicated in Figure 2. External excitation is commonly related to optical or thermal sources such as lamps, flash, or laser heating, while internal excitation is produced by eddy current, electromagnetic, ultrasonic, or vibration-based sources. Active thermography methods are classified by scanning geometry into point, line, and surface (area) scanning.

**Figure 4:**
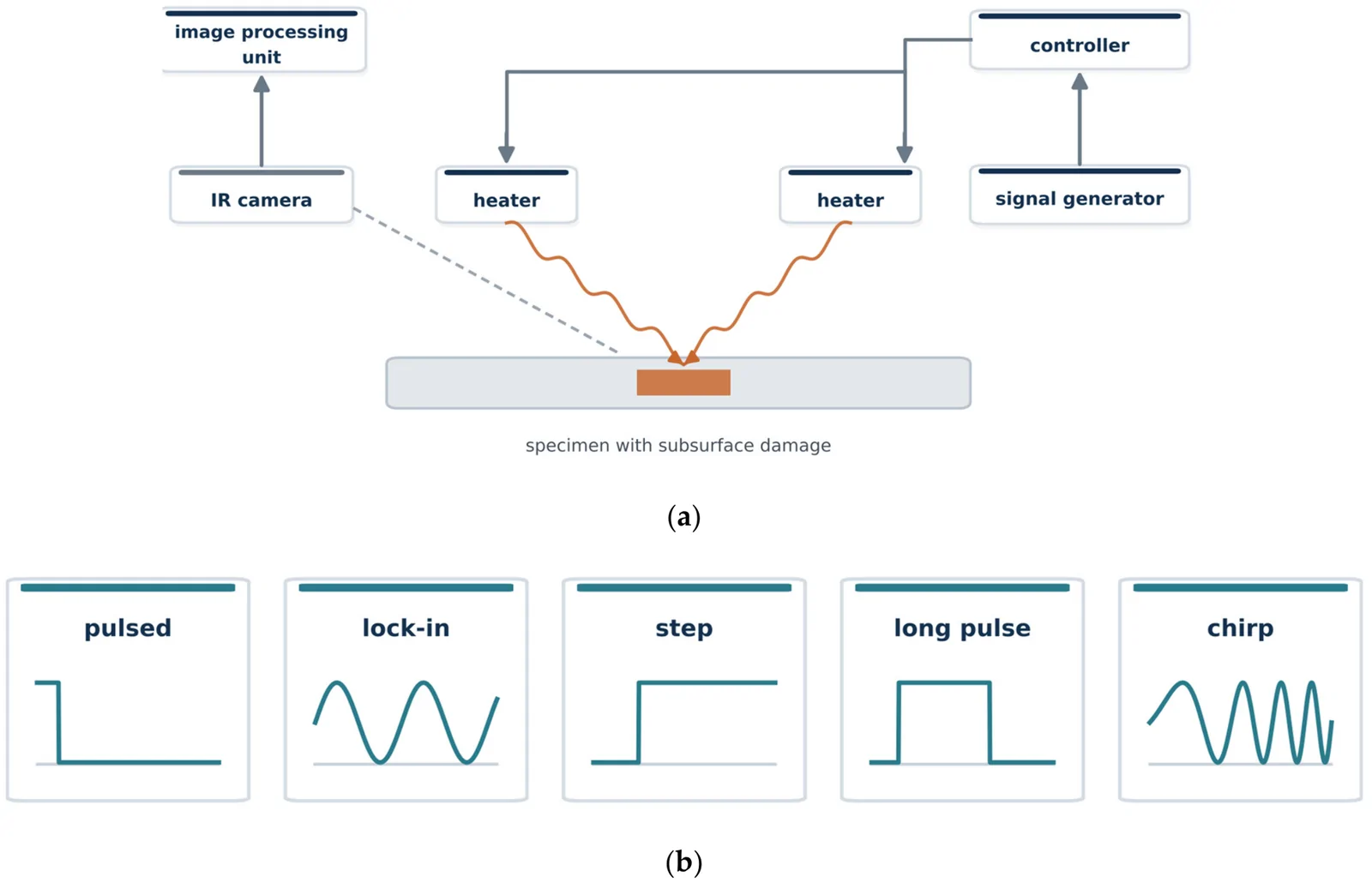
Optically excited thermography: (**a**) schematic diagram of the measurement arrangement; (**b**) heating functions or forms. Diagram drawn with the assistance of Claude (Anthropic, claude.ai); the content was specified and verified by the authors.

The main active IRT techniques include pulsed thermography (PT), square-pulse thermography (SPT), burst thermography (BT) [47], lock-in thermography (LT), step heating thermography (ST), pulsed phase thermography (PPT), square-pulse phase thermography (SPPT), burst phase thermography (BPT), and frequency-modulated thermography (FMT) [3,49]. Each of these techniques applies energy to the specimen through one of these heating functions or waveforms as shown in Figure 2 and Figure 4b, which differ primarily in the temporal profile of the excitation; that is, whether it consists of a single short pulse, a continuous periodic signal, or a frequency-swept signal.

In PT, a short heat pulse is applied to the specimen surface, and the cooling response is monitored [50]. SPT uses a square-shaped heat pulse, while BT uses a burst-type heat excitation. These methods mainly depend on the time-domain temperature response.

Phase-based methods, such as PPT, SPPT, and BPT, use the thermal response obtained from pulsed, square-pulse, or burst excitation and process it in the frequency domain. This provides phase and amplitude information, which can improve damage visibility and reduce the effect of non-uniform heating.

LT uses periodic or modulated heating and analyzes the steady periodic thermal response. FMT uses a frequency-varying excitation signal, allowing thermal information to be collected at different frequencies during one inspection.

In vibrothermography, especially self-heating or defect-resonance-based methods, heat is not applied as an external surface heat source. In such cases, heat is generated locally at damage sites due to vibration, friction, or LDR. The heating mechanism can be described as SHT, VHT, or AHT, where SHT refers to surface heating, VHT to volume heating, in which electromagnetic excitation deposits the energy throughout the thickness instead of at the surface [51], and AHT to abnormal or self-heating generated mainly at damage sites.

## 3. Optical Thermography

In optical thermography, thermal energy is introduced on the specimen surface using the external optical sources introduced in Section 2.3. As observed from Figure 2, the heating style of optical thermography is SHT. As shown in Figure 4b, the basic waveforms or heating functions are PT, LT, ST, LPT, and FMT [3]. All methods have their unique benefits based on the material characteristics, the type of damage, and the inspection limitations.

### 3.1. Pulsed Thermography

PT exposes the specimen to high-intensity heating for short periods through optical excitation sources; following the pulse, the temporal temperature response is captured with an infrared camera; the heating function of PT is shown in Figure 4b. The further progression of surface temperature after the pulse is recorded with the help of infrared cameras [43]. PT was originally proposed by Parker in 1961 [52,53]. In PT, the presence of damage changes the thermal response of the material because of local variations in the diffusion rate. Therefore, the damage depth is a concern in PT, in addition to local variation in emissivity and non-uniform heating [3].

Damage depth (z) can be estimated from the peak-slope time, which is the instant at which the derivative of the thermal contrast with respect to the logarithm of time reaches its maximum, using the relationship [3,54,55,56]:

(5) $$t_{peak}=\frac{3.64z^{2}}{\pi^{2}\alpha },$$

where $t_{peak}$ is the time at which the slope of the thermal contrast reaches its maximum. Solving Equation (5) for depth gives $z=C_{1}\sqrt{\alpha t_{peak}}$, with $C_{1}=\pi /\sqrt{3.64}\approx 1.65$. Experimental and numerical studies have shown that the peak slope time is practically independent of damage size, and the coefficient 3.64 holds regardless of the material, provided that z/L≤0.5 [55]. The distinction matters because the two instants are not interchangeable. The time of maximum thermal contrast depends on the lateral size of the damage and on the sound reference area, whereas the peak-slope time depends on the one-dimensional cooling transient alone, for which the coefficient 3.64 was derived. Using the peak-contrast time in Equation (5) overestimates the depth.

The equivalent estimate in the frequency domain is obtained from PPT (Section 3.4). Instead of a characteristic time, this approach uses the blind frequency, i.e., the frequency at which the phase contrast between the damaged and the sound region vanishes; below this frequency the damage produces a detectable phase contrast. The method is based on the thermal diffusion length, $\mu =\sqrt{\alpha /\pi f}$, and the damage depth is estimated from Equation (6) [55,56,57]:

(6) $$z=C_{2}\cdot \mu =C_{2}\sqrt{\frac{\alpha }{\pi f_{b}}},$$

where $C_{2}$ is an empirical correlation constant and $f_{b}$ (Hz) is the blind frequency. When the thermal properties of the material are known, the quantitative PPT analysis therefore requires only the determination of $f_{b}$ and $C_{2}$. PPT results [57] agreed with these numbers for homogeneous materials: $C_{2}$ ≈ 1.72 for steel, and $C_{2}$ ≈ 2 for Plexiglas, with $C_{2}=1.82$ being the value most commonly adopted in experimental studies [55]. The inversion problem reduces then to the estimation of $f_{b}$. This relationship shows that deeper damage results in delayed thermal signatures following the pulse. Although PT is valued for rapid inspection, it is sensitive to surface emissivity variations and susceptible to non-uniform heating, which constrains the effectiveness of PT on thick composites [53].

The surface temperature decay after an ideal Dirac pulse is given by Equation (7):

(7) $$T\left(t\right)=\frac{Q}{e\sqrt{\pi t}},t>0,$$

where Q is the energy pulse density, and e is the thermal effusivity, which can be obtained as in Equation (8):

(8) $$e=\sqrt{k\rho c_{p}}.$$

Equation (7) is used to correlate measured cooling curves with intrinsic thermal properties, which are essential to interpreting thermograms as well as to relating a surface temperature evolution to subsurface features. Two assumptions behind Equations (5)–(9) require qualification for laminated composites. The relations assume one-dimensional diffusion in a homogeneous, isotropic, semi-infinite medium, whereas a PMC laminate is neither homogeneous nor isotropic: 6.80 and 0.32 W m^−1^ K^−1^ are reported along and across the fibres for a laminate of 34% fibre volume fraction [58]. Heat, therefore, spreads along the plies more readily than through the thickness, so a defect appears wider and shallower than it is and the inverted depth is biased. The semi-infinite assumption also fails once the thermal front reaches the rear face, which for thin laminates occurs within the observation window. The coefficients in Equations (5), (6) and (9) should, therefore, be read as calibration constants for a given material and lay-up, and every depth quoted here is reported with its laminate. Where absolute property values are required, inverse methods that fit a three-dimensional anisotropic model to the recorded temperature field replace the closed-form relations [58,59].

### 3.2. Lock-In Thermography

In LT, excitation is a continuous sinusoidal wave at a single frequency [60]. The heating function of LT is shown in Figure 4b. The phase lag or shift between the input thermal wave and the output or reflected thermal wave is used for the identification of damage [53]. In periodically modulated heating, the effective probing depth of the technique is determined by the thermal diffusion length, which sets the frequency-depth trade-off of lock-in techniques [38,61,62]. The advantage of LT is that the phase shift is insensitive to local variation of emissivity compared to PT [3]. However, detecting flaws at various depths requires repeating the test at several frequencies to avoid blind frequencies, which makes the method time-consuming. In addition to sweeping multiple lock-in frequencies, combining the phase images and applying processing algorithms best suited to lock-in data, such as binarization, morphological operations, and multivariate outlier analysis, are also good choices. The thermal diffusion length ϕ is:

(9) $$\phi =\sqrt{\frac{\alpha }{\pi f}}=\sqrt{\frac{2\alpha }{\omega }}.$$

Therefore, the choice of modulation frequency directly controls how deep the technique can effectively probe into the material.

For a semi-infinite homogeneous medium subjected to sinusoidal surface heating, the steady periodic solution of Equation (2) is a strongly damped thermal wave [62,63,64,65]:

(10) $$T\left(z,t\right)=T_{0}\exp\left(-\frac{z}{\phi }\right)\cos\left(2\pi ft-\frac{z}{\phi }\right).$$

Higher frequencies localize thermal waves near the surface and therefore improve the detection of shallow damage, while the lower frequencies introduce larger diffusion lengths and therefore detect deeper damage. LT provides better noise reduction, insensitivity to surface emissivity variation, and quantitative depth profiling, and is recommended for detecting debonding and impact damage [66,67,68,69]. The acquisition times are, however, longer, and signal processing is more complicated [69,70].

### 3.3. Step Heating and Long Pulse Thermography

ST uses a prolonged heat flux over long intervals to allow more penetration, which makes it useful in large or low-diffusivity materials [71]. LPT records thermal signals in the cooling process after a long period of heating [43]. While ST gets data during heating, LPT measures it during cool-down. The main shortcoming of LPT is that it is susceptible to noise in the environment, and in most cases, it generates low-contrast images, which can be improved by using advanced signal processing [72]. The heating function of ST and LPT is shown in Figure 4b.

Homogeneous 1D heat diffusion response to a step-pulse heating source $Q_{0}$ can be represented as [73,74,75,76]:

(11) $$T\left(0,t\right)=\frac{2Q_{0}}{e}\sqrt{\frac{t}{\pi }},\;t>0.$$

For the LPT case, the same solution applies, but the rise and cool-down phases are usually treated separately: Equations (12) and (13) are obtained by applying the superposition principle to Equation (11), the same one-dimensional heat diffusion solution used in step heating [36,43]. The square pulse is modeled as a step on at t=0 and step off at $t=\tau_{p}$.

(12) $$\Delta T_{\mathrm{rise}}\left(0,t\right)=\frac{2Q_{0}}{k}\sqrt{\frac{\alpha t}{\pi }},0<t<\tau_{p},$$

(13) $$\Delta T_{\mathrm{cool}}\left(0,t\right)=\frac{2Q_{0}}{k}\sqrt{\frac{\alpha }{\pi }}\left(\sqrt{t}-\sqrt{t-\tau_{p}}\right),t\geq \tau_{p}.$$

An experimental study by Nategh [76] showed that ST is efficient on low-thermal-diffusivity materials, whereas pulse thermography is more efficient on materials with high thermal diffusivity. The choice of technique is therefore based on the material properties, the damage type, and the operational constraints.

### 3.4. Pulsed Phase Thermography

PPT is an NDT method that combines the advantages of PT and LT with frequency-based analysis. A short thermal pulse is applied to the material, and the cooling response is recorded and converted into the frequency domain using the Discrete Fourier Transform (DFT).

Damage can be detected at various depths due to differences in the frequency of the radiation used; PPT can detect low frequencies at greater depths and high frequencies at shallower depths. The use of phase images is particularly valuable since they diminish the impact of differential heating and surface reflections, thus revealing damage. In general, PPT is a successful method of ascertaining subsurface damage in composite materials, aerospace structures, etc.

In practice, PPT processing typically proceeds pixel-by-pixel: the recorded temperature decay at each surface point is transformed via the DFT into an amplitude and a phase spectrum, and the phase image at a given frequency is retained for analysis because, unlike the raw amplitude, phase is largely insensitive to non-uniform heating, local emissivity variation, and surface reflections, all of which otherwise degrade raw PT contrast. Because deeper damage responds preferentially to lower blind frequencies and shallower damage at higher ones, examining the phase image across a range of frequencies from a single pulsed acquisition allows an approximate depth range to be probed without repeating the experiment, unlike LT, which requires a separate acquisition per frequency. This computational efficiency, combined with improved contrast, is the main reason PPT is often regarded as combining the strengths of PT and LT, although it inherits the finite penetration depth and limited pulse energy constraints of PT, and its frequency resolution is limited by the total acquisition duration.

### 3.5. Frequency Modulated Thermography

FMT uses linearly swept chirp excitation, better probing a depth range in a single measurement [76]. This method overcomes the limited depth resolution of fixed-frequency LT methods [77]. Pulse-compression correlation processing with FMT offers enhanced depth resolution and signal-to-noise ratio, without the need for the high peak power required by pulsed methods [78,79].

The chirp signal used in FMT is typically designed with a frequency range and sweep duration selected according to the thermal diffusivity and expected damage depth range of a MUT, since the lowest frequency in the sweep determines the maximum achievable probing depth, analogous to the frequency selection constraint in LT. After acquisition, the recorded thermal response is cross-correlated with a reference chirp, a pulse-compression step that concentrates the signal energy in time and improves the signal-to-noise ratio relative to using any single frequency component alone. FMT has been demonstrated on both metallic and PMCs, and its main practical advantage over LT is that a single chirp acquisition replaces the multiple fixed-frequency acquisitions that LT would otherwise require to cover the same depth range, at the cost of a more demanding excitation source capable of accurately reproducing the swept-frequency waveform. The advantages of FMT are, however, bounded in practice. Pulse compression recovers signal-to-noise ratio only when the sweep is long compared with the thermal time constant of the inspected depth, so acquisition time is set by the deepest feature and is not shorter than a lock-in measurement at the corresponding frequency. The chirp amplitude is capped by the glass-transition limit of Section 2.2. The low-frequency end of the sweep carries the depth information but is most affected by convective losses and ambient drift. The correlation peak depends on the window and reference used, so FMT depth estimates transfer between laboratories less readily than the blind-frequency estimate of PPT. The three frequency-domain modalities therefore differ: lock-in thermography gives the highest signal-to-noise ratio at a single interrogation depth, at one acquisition per frequency; PPT gives the whole phase spectrum from one pulsed acquisition and supports Equation (6), but inherits high peak power and spectral leakage; FMT covers a depth range in one acquisition at moderate power, which suits thick or unknown-thickness laminates, at the cost of the longest measurement time per depth decade and the most involved processing.

While the optically excited techniques described above rely on an external heat source and well-established diffusive depth-inversion models (Equations (5), (6) and (9)), they share an inherent limitation for PMCs: heat must first diffuse from the surface to the damage and back before a contrast can be observed, which constrains detectability of deep or weakly reflective interfaces and can be affected by surface emissivity and non-uniform heating, as noted for PT and LT above. SHVT (see Section 4.3) addresses this limitation by generating heat internally, exploiting the intrinsic viscoelastic hysteresis of the polymer matrix under cyclic mechanical loading rather than surface heat diffusion [13,80,81,82]. Because the heat source is localized at or near the damage itself, SHVT can achieve strong damage-to-background thermal contrast for interfacial or embedded damage that is comparatively difficult to detect optically. However, the level of quantification achieved by SHVT and related vibrothermographic methods remains less mature than that of PT or optical LT: no depth-inversion relationship analogous to Equation (5) has yet been validated to an equivalent degree, and the results depend more strongly on the viscoelastic properties, the geometry, and the boundary conditions of the specific structure under test [12]. SHVT is therefore generally selected over optical excitation not as a replacement but as a complementary technique, most valuable when access is restricted to one side of the structure, when the damage is not amenable to purely diffusive detection, or when large-area, low-power inspection is required, as discussed in detail in Section 4.

### 3.6. Application Scenarios and Complementarity with Conventional Methods

The modalities above have been described by their heat-transfer behaviour. Their place in practice follows from two properties that the conventional methods do not share: the measurement is made without contact, and over a field rather than at a point. Both matter for composites, whose properties vary with position because of the lay-up and the local fibre volume fraction, and whose service condition is of interest while the part is loaded.

Active thermography is therefore not restricted to finding damage. The same acquisitions also carry the thermal transport properties of the material. Thermal effusivity follows from the early cooling transient through Equation (8) and has been used to image porosity in CFRP [83]. In-plane diffusivity and conductivity follow from the spatial decay of the modulated response in laser-line lock-in thermography [84]. Full conductivity tensors have been recovered for carbon-fibre/epoxy by fitting a three-dimensional model to a thermographically recorded, laser-induced temperature field, giving 6.80 against 0.32 W m^−1^ K^−1^ along and across the fibres [58]. Related inverse formulations work from a measurement with source and detector on the same side, accounting for the finite dimensions and real shape of the specimen [59]. Beyond transport properties, micro-scale thermography with machine learning has been used to quantify filler dispersion and follow the state of cure [85], and the local dissipation measured by SHVT is a spatially resolved analogue of the loss modulus obtained in bulk by dynamic mechanical analysis.

Against this stand, the laser flash method, the guarded hot plate and the heat flow meter, which remain the reference techniques and are more accurate on a homogeneous specimen [86]. They are offline, require a machined specimen, are limited to small geometries, and return a single value for the whole specimen. Thermographic methods are therefore complementary rather than substitutes: the conventional methods calibrate, and the thermographic methods extend the measurement to the full field, the assembled component and the loaded state. Table 2 sets out that division for each modality treated above.

One application outside damage detection deserves separate mention. Polymers doped with thermochromic microcapsules or with vanadium-dioxide particles change their optical or infrared response at a transition temperature, and are being developed for adaptive thermal management, temperature indication and camouflage [87]. Their performance is limited by the low thermal conductivity of the polymer matrix: heat reaches the active particles slowly and leaves them slowly, so the switching and recovery of the colour change lag behind the applied thermal load, and the lag grows with the thickness of the film. Infrared thermography suits this problem because it measures the surface temperature field without contact at the frame rates on which the switching occurs, so the thermal transient can be observed together with the optical transition; it has already been used to confirm the heat-retention behaviour of polyurethane films filled with thermochromic dye microcapsules [88]. Step heating or long-pulse excitation gives the switching and recovery times, and lock-in thermography the through-thickness diffusivity that governs them, which together separate the response time of the agent from the transport delay of the matrix.

**Table 2:** Application scenarios of the thermographic modalities treated in Section 3 and Section 4, the quantity each one measures, the state in which it can be applied, and the conventional method it complements.

Modality	Measurable Quantity	Operating State	Conventional Method Complemented
Pulsed thermography (PT) [52,54,55,83]	Presence, in-plane extent and depth of subsurface damage; thermal effusivity and diffusivity from the cooling transient	Offline, unloaded; single-sided; seconds per field of view	Laser flash analysis, which gives diffusivity as a single bulk value on a small, machined coupon
Lock-in thermography (LT) [61,63,66,84]	Phase contrast at a selected interrogation depth; in-plane diffusivity and conductivity from the phase slope	Offline or in service; compatible with a loaded specimen, so property change can be tracked during fatigue pauses or axial tension	Guarded hot plate and heat flow meter, which give a steady-state bulk conductivity without spatial resolution
Step heating and long pulse thermography (ST, LPT) [71,72,74,75]	Remaining wall thickness and slow transients in thick laminates	Offline; low power, large area	Ultrasonic thickness gauging, which is point-wise and needs couplant
Pulsed phase thermography (PPT) [55,56,57]	Depth from the blind frequency; the whole phase spectrum from one acquisition	Offline, unloaded; single-sided	Destructive sectioning and micrography for depth confirmation
Frequency modulated thermography (FMT) [77,78,89]	A range of depths in a single sweep at moderate peak power	Offline; suited to thick or unknown-thickness parts	Multi-frequency lock-in acquisition, one frequency at a time
Ultrasonic vibrothermography (UT) [45,90,91,92]	Presence and location of closed, friction-active cracks and disbonds	Offline; contact excitation; seconds per excitation	Penetrant testing, which reaches only surface-breaking defects
Local defect resonance (LDR) [93,94]	Location of damage that supports a local resonance, at low excitation power	Offline; contact; requires an auxiliary vibrometry scan to find the frequency	Laser Doppler vibrometry used alone, which maps vibration but not dissipation
Self-heating based vibrothermography (SHVT) [82,95,96,97]	Presence, in-plane extent and relative depth of matrix-dominated damage; local viscoelastic energy dissipation	Offline or during cyclic loading; single-sided; surface temperature rise below about 2 °C	Dynamic mechanical analysis, which gives the loss modulus as a bulk average on a small specimen

## 4. Vibrothermography

Vibrothermography was originally introduced in the late 1970s [3,98]. In vibrothermography, optical excitation is replaced by vibration or elastic waves, whereby mechanical or acoustic energy is converted into localized heating. These localized temperature variations on the surface of the material are detected by infrared cameras to reveal flaws hidden within a MUT [89].

The excitation is normally applied with instruments such as ultrasonic transducers, shakers, or acoustic horns. The temperature rise is caused by several mechanisms, which include frictional heating, viscoelastic dissipation, heating caused by plastic deformation, and thermoelasticity in certain cases. Frictional heating is caused by microscale sliding of asperities on closed cracks, while plastic deformation heating is caused by crack-face impact of open cracks or partially open cracks. The source of thermoelastic heating is the elastic deflection of flawless parts of metals or polymers.

The benefits of vibration-based methods are that they produce heat internally at damage sites, which is possible at low power and with a minimal thermal gradient. These features make them especially useful with cracks, flat-bottom defects (FBD), BVID, and kissing bonds [81].

**Figure 5:**
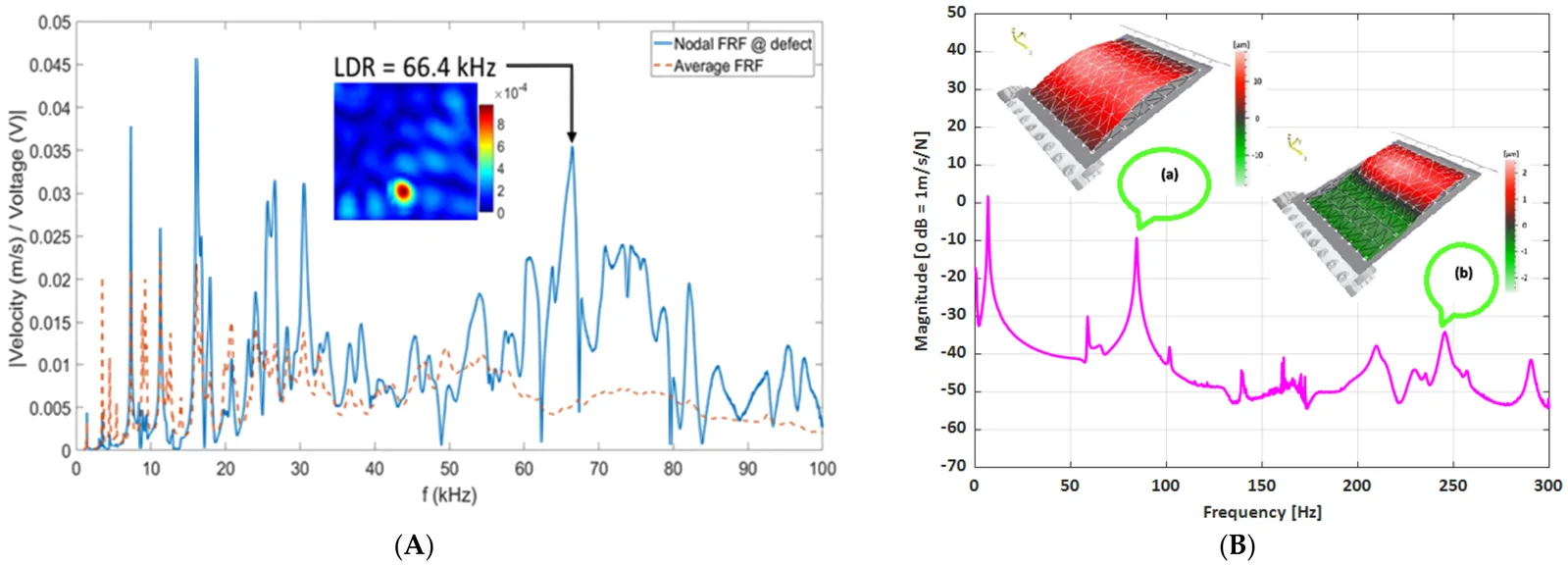
(**A**) Frequency response function (FRF) with a selected defect-specific frequency of a CFRP sample with an FBD [99]; (**B**) FRF of a 1 mm GFRP plate containing an FBD, the insets, labelled (a) and (b) in the reproduced original, show the mode shapes measured by scanning LDV at the two marked resonances, the first bending mode near 85 Hz and the fifth bending mode near 245 Hz, reproduced from [100].

Internal damping prevalent in PMCs is characterized by viscoelastic dissipation, which is more dominant at low power or low-frequency vibration. In the recently emerging vibrothermography techniques, excitation can be applied in two ways: at the natural frequency of the structure or MUT, which also depends on the boundary conditions (the case of SHVT), and at a defect-specific resonance (the case of LDR). Using these approaches, different responses are obtained: a temperature distribution in the case of SHVT, or a change in the frequency response, either of which enables the anomaly to be detected. The difficulty is greater in the case of LDR, because identifying the specific resonance of the damage is harder than exciting the whole geometry. As shown in Figure 5, the excitation of LDR and SHVT is different, and their objectives are also different: LDR aims to excite the damaged region, whereas SHVT excites the whole structure. One of the goals of SHVT is to achieve a better distribution of heat across the structure so that the entire region is covered, since in practice the location of the damage is unknown.

### 4.1. Ultrasonic Vibrothermography

**Figure 6:**
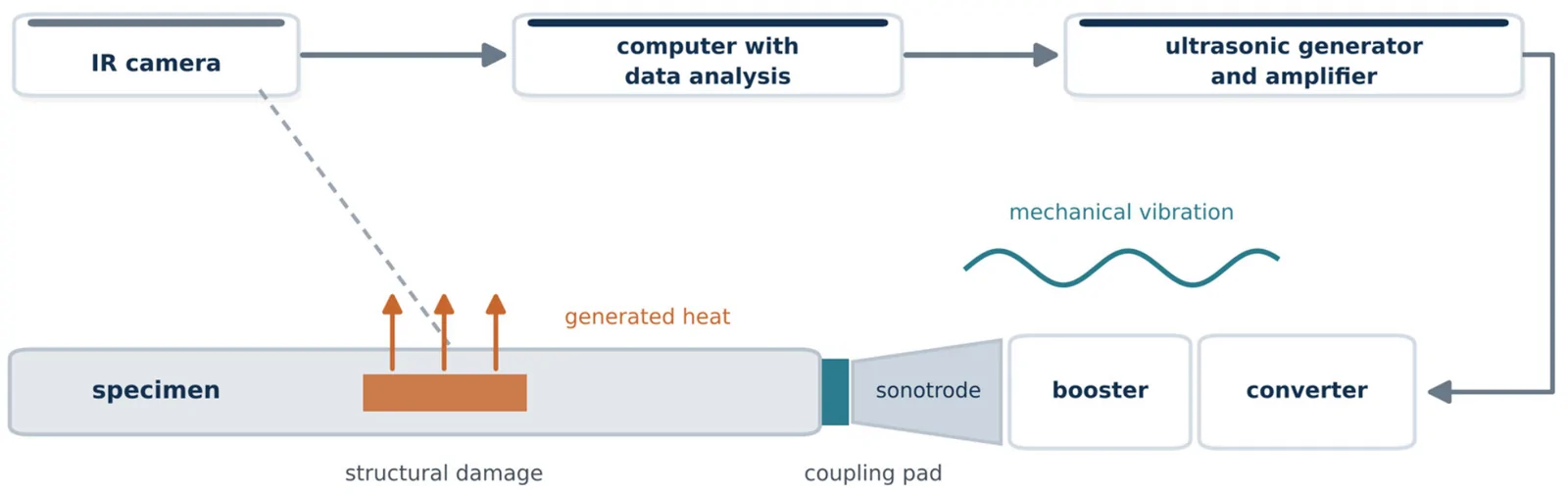
Operational principle of ultrasonic vibrothermography. Diagram drawn with the assistance of Claude (Anthropic, claude.ai); the content was specified and verified by the authors.

Ultrasonic excitation is used to produce heat by friction and crack-clapping mechanisms. Intermittent contacts or sliding on the crack faces due to high-frequency vibration are created, thus creating intense local heating effects, and thermoelastic effects are insignificant. Studies consistently identify friction and clapping as the predominant processes that induce observable temperature variations in high-frequency excited materials [48]. Figure 6 shows the principles and components of ultrasonic vibrothermography.

Ultrasonic vibrothermography (thermosonics) utilizes intense ultrasonic vibrations to augment frictional rubbing and plastic deformation at discontinuities [45,90]. Common variants of ultrasonic vibrothermography are ultrasound lock-in thermography, ultrasound burst thermography, and ultrasound burst phase thermography, which vary in excitation waveform or heating function [4,48,91].

Modern studies are aimed at reducing the amount of mechanical energy to provide or ensure truly non-destructive operation and boosting area coverage. Among the methods to achieve this, the process of choosing excitation frequencies that are close to defect resonance, choosing optimum parameters to excite, reducing the amount of power input, and preserving the heating effects is one of the approaches [91,92]. Where friction dominates, the local heat flux generated at a sliding interface is estimated as:

(14) $$q=\mu_{f}\sigma_{N}|v_{\tau }|,$$

where $\mu_{f}$ is the friction coefficient, $v_{\tau }$ is the relative velocity vector, $\sigma_{N}$ is the normal stress of the interface.

In cases where friction prevails, the heat generation flux can also be expressed in a more detailed way:

(15) $$q\left(t\right)=\left[\mu_{d}+\left(\mu_{s}-\mu_{d}\right){exp}^{-\Psi |v_{\tau }\left(t\right)|}\right]\cdot \sigma_{N}\left(t\right)\cdot v_{\tau }\left(t\right),$$

where q(t) is the heat flux generated, $\mu_{d}$ and $\mu_{s}$ are dynamic and static friction coefficients, Ψ is a number related to the velocity at which the transition from static to dynamic friction occurs, and the remaining symbols are as defined for Equation (14).

Ultrasound lock-in thermography operates on the principle of cross-correlating a periodically modulated thermal excitation signal with a high-frequency electronic reference to extract amplitude and phase information from the infrared detector [61]. Subsurface discontinuities such as delaminations, voids, or disbonds in fiber-reinforced polymer composites introduce additional thermal resistance that manifests as a localized phase shift superimposed on the baseline phase lag between the reference signal and the damage-free thermal response.

### 4.2. Local Defect Resonance

LDR-frequency excitation is a markedly more effective method of contrasting damage without high ultrasonic power input [12]. LDR techniques can be used with LT or frequency-modulated excitation to improve sensitivity [93,101].

The overall concept is to make the structure vibrate at LDR, so that the damaged region vibrates with significantly higher amplitude than the surrounding healthy material, and thus, the structure can be heated effectively. In addition to standard out-of-plane LDR, in-plane LDRs (motion parallel to the surface) are found to be especially effective for composites, producing strong tangential rubbing at the damage faces and excitation at higher frequencies, giving significantly stronger thermal contrast with low-power piezoelectric excitation [91,94].

The LDR frequency for a circular defect can be approximated from its effective stiffness ($K_{\mathrm{eff}}$) and mass ($M_{\mathrm{eff}}$) as in Equation (16) [15]:

(16) $$f_{\mathrm{LDR}}=\frac{1}{2\pi }\sqrt{\frac{K_{\mathrm{eff}}}{M_{\mathrm{eff}}}}.$$

In the case of FBDs, these parameters are material-dependent and defect-dependent, which allows optimal excitation frequencies to be predicted theoretically.

(17) $$K_{\mathrm{eff}}=\frac{192\pi B}{R^{2}},M_{\mathrm{eff}}=1.8m,\mathrm{where}\;m=\pi \rho hR^{2},\;B=\frac{Eh^{3}}{12(1-\nu^{2})}.$$

By combining these, the resonant frequency can be approximated as:

(18) $$f_{\mathrm{LDR}}\approx 1.64\frac{h}{R^{2}}\sqrt{\frac{E}{12\rho (1-\nu^{2})}}.$$

The numerical prefactor follows directly from Equation (17), since $\frac{{\left(192/1.8\right)}^{1/2}}{2\pi }$ = 1.64; the value 1.6 quoted in parts of the literature [93,94] is a rounding of the same result. Equation (18) shows that a stiffer material (larger E) and a lower density ρ raise the frequency of resonance, that a larger defect radius R lowers it quadratically, and that a greater thickness h of the delaminated layer raises it linearly. The dependence on h is frequently misstated: because $f_{\mathrm{LDR}}$ ∝ $\frac{h}{R^{2}}$, thinner delaminated layers resonate at lower frequencies, not higher ones. Poisson’s ratio ν modifies the frequency only weakly through the factor $\left(1-\nu^{2}\right)$. It is noted that Equation (18) assumes a fully clamped, circular, single-layer delamination of uniform thickness, and should therefore be used to bracket a frequency sweep rather than as a predictive design formula for arbitrary defect shapes.

### 4.3. Self-Heating Based Vibrothermography

**Figure 7:**
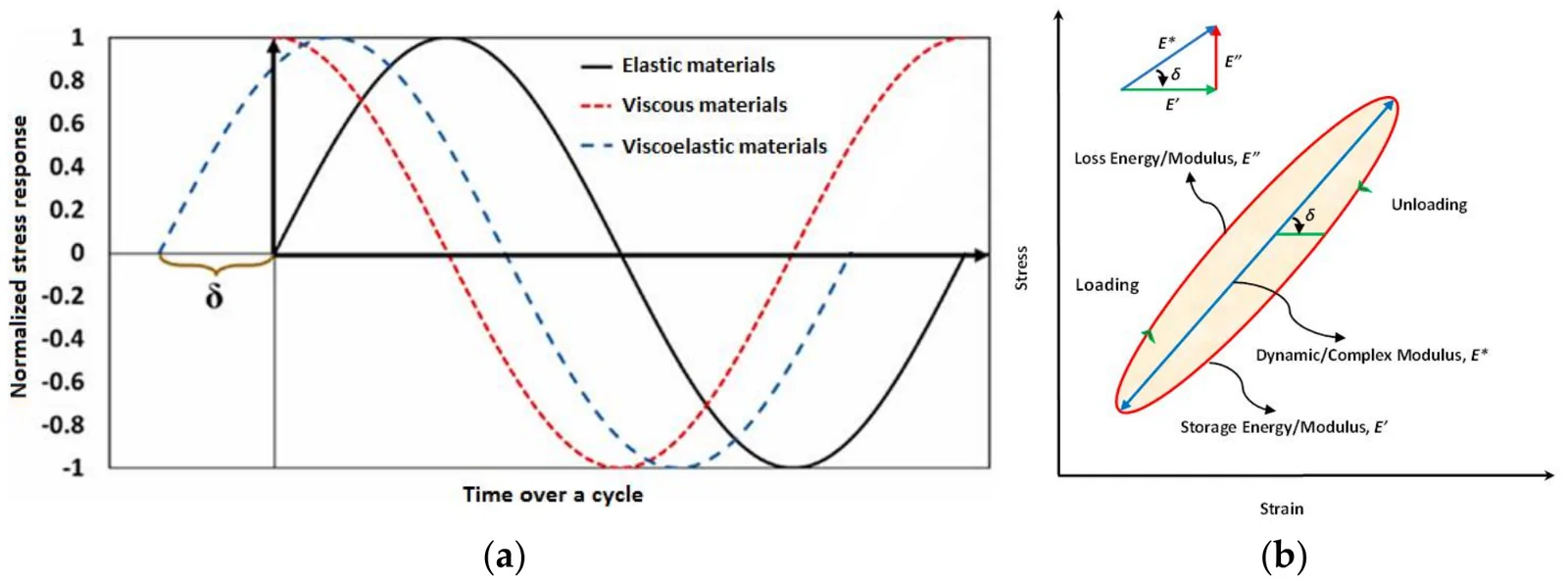
(**a**) Normalized stress response to cyclic strain-driven loading for elastic, viscous, and viscoelastic materials, defining the phase lag δ; Reproduced from [103]. (**b**) The resulting hysteresis loop, whose major axis gives the complex modulus $E^{*}$, whose lower and upper boundaries correspond to the storage and the loss modulus, and whose enclosed area is the energy dissipated per cycle [103].

SHVT is a non-destructive inspection technique that integrates IRT with the heat generated internally by the viscoelasticity of the material under multi-harmonic vibration. In contrast to existing thermographic techniques, which rely on the use of external sources of heat, SHVT takes advantage of the thermomechanical nature of resonant vibration or multi-harmonically excited structures. When PMCs are excited at natural vibration frequencies, cyclic strains generate heat proportional to excitation frequency and material loss modulus due to viscoelastic hysteresis [102]. Figure 7a shows that there is a time lag δ between stress and strain, which gives rise to the frequency and temperature dependent storage $E^{\prime }$ and loss moduli $E^{\prime \prime }$. The sum of both moduli is the complex modulus $E^{*}$ as a function of frequency and temperature when the load is applied sinusoidally.

**Figure 8:**
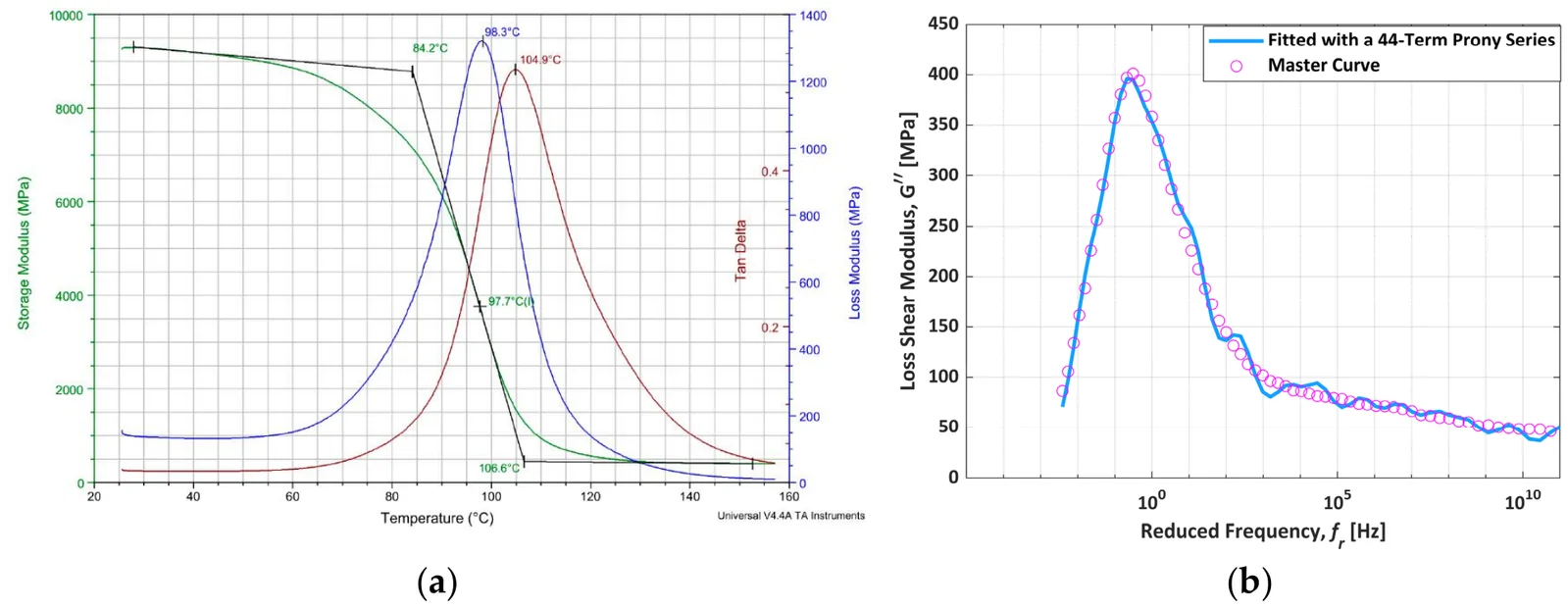
Measured viscoelastic response of glass/epoxy laminates: (**a**) storage modulus, loss modulus, and tan δ against temperature from dynamic mechanical analysis (DMA) [103]; (**b**) loss shear modulus against reduced frequency, the master curve constructed by time-temperature superposition with its 44-term Prony series fit [104].

For a generalized Maxwell model with n relaxation modes, the storage and loss moduli are

(19) $$G^{\prime }\left(\omega ,T\right)=\sum_{i=1}^{n}\left(\frac{G_{i}(T)(\omega \tau_{i}{)}^{2}}{1+(\omega \tau_{i}{)}^{2}}\right),$$

(20) $$G^{\prime \prime }\left(\omega ,T\right)=\sum_{i=1}^{n}\left(\frac{G_{i}\left(T\right)\omega \tau_{i}}{1+(\omega \tau_{i}{)}^{2}}\right),$$

where G′ is the storage modulus, representing elastic energy stored during cyclic deformation; G″ is the loss modulus, representing viscous energy dissipated during cyclic deformation; ω is the angular frequency of oscillation; n is the number of relaxation modes considered; $G_{i}\left(T\right)$ the modulus associated with the ith mode; and $\tau_{i}$ its relaxation time. Both moduli are also illustrated in Figure 8.

In practice, G′ and G″ are not known a priori. They are measured by torsional oscillatory rheometry over a range of temperatures and frequencies, collapsed onto a master curve through the time-temperature superposition principle with a shift factor $a_{T}$ [105], and then represented by an n-term Prony series whose coefficients are obtained by minimizing the residual against the measured storage modulus, loss modulus and loss factor, simultaneously. Because Equations (19) and (20) supply the material input to the heat generation term, the accuracy of the Prony fit propagates directly into any predicted self-heating field.

When composite structures are excited at their natural vibration frequencies, the rate of heat generation is proportional to the excitation frequency and to the loss modulus of the material, through the viscoelastic hysteresis of the cyclic strain. The rate of heat generation per unit volume (${\dot{q}}_{gen}$) in cyclic stressing enters the energy balance of Equation (21) and is given explicitly by Equation (22) [106,107]:

(21) $$\int \left(\rho c_{p}\frac{\partial T}{\partial t}\right)dV+\left[\int \left(-k\nabla^{2}T\right)dV+\int \eta \left(T-T_{a}\right)dA_{cv}+\int \varepsilon \beta \left(T^{4}-T_{a}^{4}\right)dA_{rd}\right]=\int {\dot{q}}_{gen}dV,$$

where V is the control volume, $A_{cv}$ and $A_{rd}$ are the surface areas exposed to convection and radiation, respectively, ε is the emissivity, and β is the Stefan-Boltzmann constant. The thermal conductivity k is assumed to be independent of temperature and thermally isotropic (i.e., $k_{x}=k_{y}=k_{z}=k$), as specified in the manufacturer’s datasheet [108]. Here, η is the convective heat transfer coefficient and $T_{a}$ the ambient temperature.

(22) $${\dot{q}}_{gen}=\frac{\omega }{2\pi }\int_{0}^{2\pi }\sigma (t)\frac{d\varepsilon (t)}{dt}\to {\dot{q}}_{gen}=3\pi f\varepsilon_{0}^{2}G^{\prime \prime }(f,T),$$

where f is the excitation frequency (Hz), $\varepsilon_{0}$ is the strain amplitude. Equation (22) shows that heat generation is directly proportional to the excitation frequency and to the square of the strain amplitude, which explains why damaged regions with locally elevated strain concentration produce disproportionately enhanced thermal signatures [109].

The numerical factor follows from the cyclic bending formulation of Dinzart et al. [106] and is retained in the self-heating models applied to PMC laminates under resonant plate excitation [104]. The corresponding expression for uniaxial cyclic loading differs from Equation (22) and is written in terms of the tensile loss modulus, ${\dot{q}}_{gen}$ = $\pi f\varepsilon_{0}^{2}E^{\prime \prime }$ [103]; the two forms are not alternatives to be chosen between, but follow from different loading geometries, and Equation (22) is the one appropriate to the bending-dominated resonant excitation used in SHVT. Because $G^{\prime \prime }$ is itself a function of frequency and temperature, Equation (22) is coupled to the energy balance of Equation (21) through the instantaneous temperature field: the heat source strengthens as the specimen warms, so the thermal response is obtained by iterative solution rather than in closed form. In numerical implementations of this coupling, radiative heat flux proved to be a critical term in the surface boundary condition rather than a second-order correction, and omitting it degrades the predicted temperature field appreciably [104].

The main benefits of SHVT are large-scale area excitation, low temperature increase that does not affect structural integrity, and the possibility to explore parts with restricted access or single-side access [110]. The detection capability of the technique depends on damage size, specimen thickness, and the viscoelastic behavior of the matrix system, and is assessed against the reported experimental evidence in Section 4.4 [106,110,111,112].

**Figure 9:**
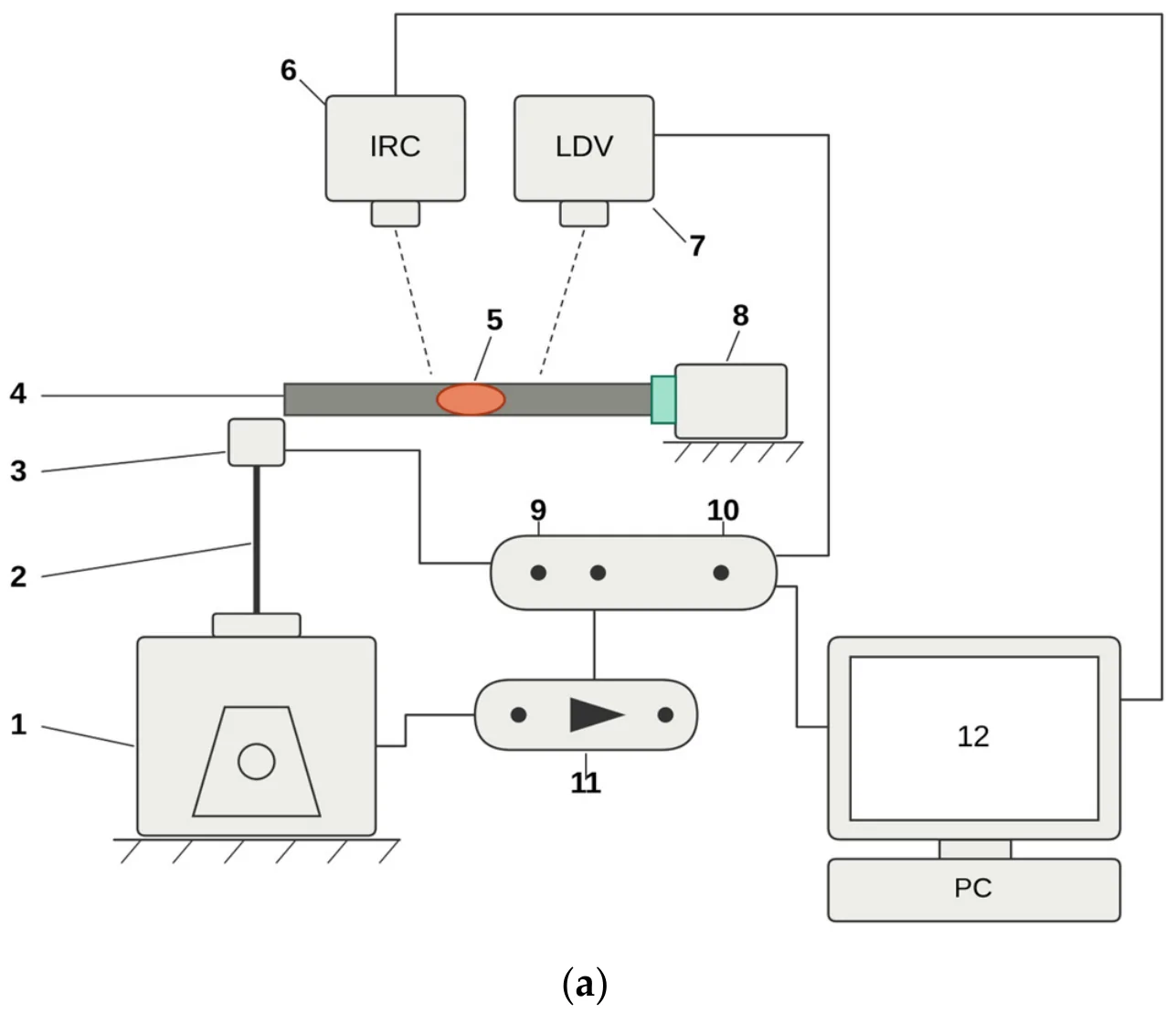

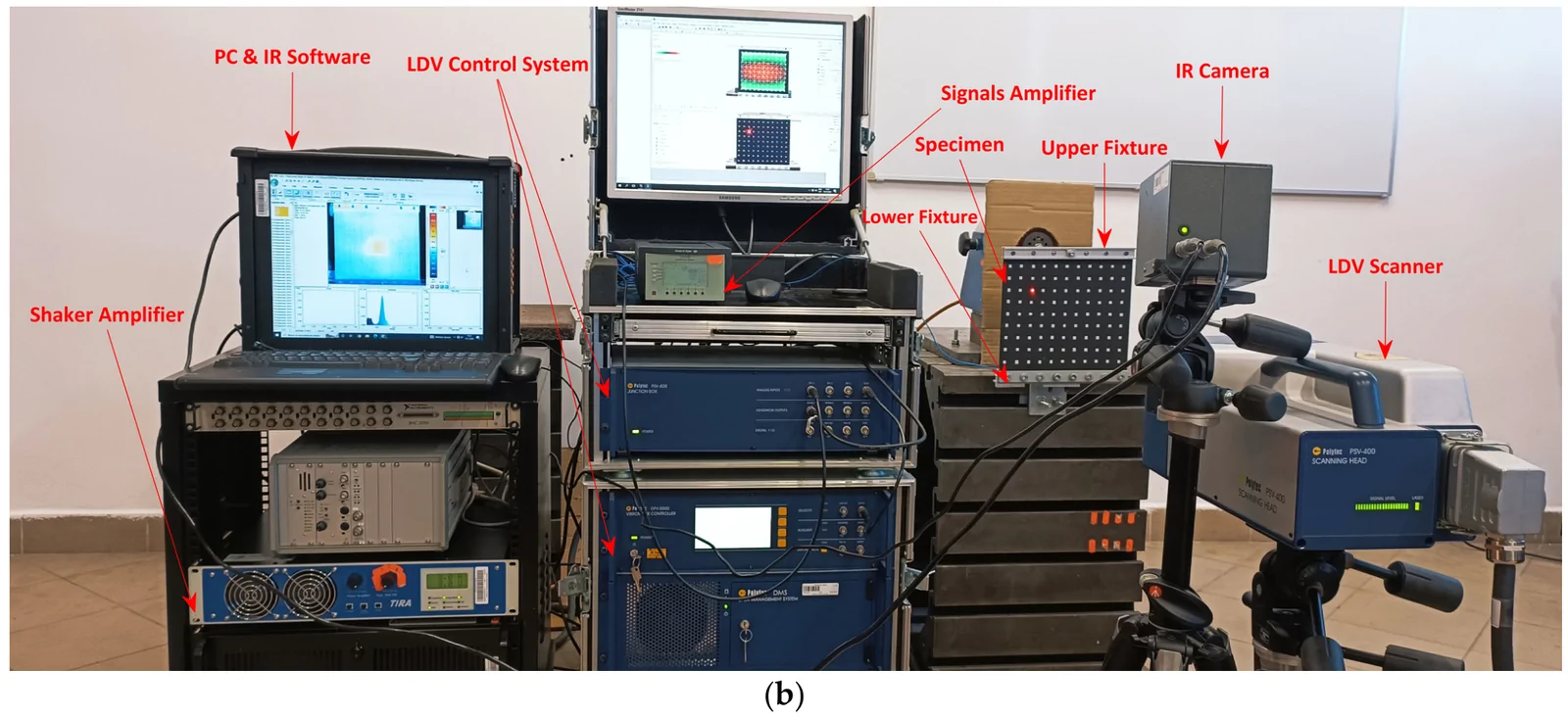
SHVT measurement system: (**a**) schematic: 1—electrodynamic shaker; 2—stringer; 3—force sensor; 4—composite plate specimen; 5—introduced damage; 6—infrared camera; 7- LDV; 8—rigid fixture with thermal insulator; 9—signal conditioner; 10—controller and data acquisition unit; 11- power amplifier; 12—PC with acquisition and processing software; drawn based on [113]; (**b**) main view of the experimental setup used for SHVT testing rig [96].

A key advantage of SHVT is that it can be used in situations where external heating cannot be applied, which makes it well suited to in-situ inspection. A schematic of the SHVT working principle is shown in Figure 9a, while its hardware implementation in Figure 9b. The operating principle of SHVT is based on thermal excitation through resonant vibration or applying external mechanical vibrations with resonant frequencies from a low-frequency range. The amount of energy dissipated in self-heating is proportional to the resulting stresses, creating localized temperature increases; multi-harmonic signal excitation is a preferred method that excites the whole structure at several resonance frequencies simultaneously [95]. Multiple resonance frequencies are used in order to excite different regions of the structure and thus obtain a better heat distribution for locating the damage [95].

Although these techniques still require improvement in terms of efficiency and simplicity under real conditions, the localization of damage with low magnitude of excitation and minimum temperature is achieved.

**Figure 10:**
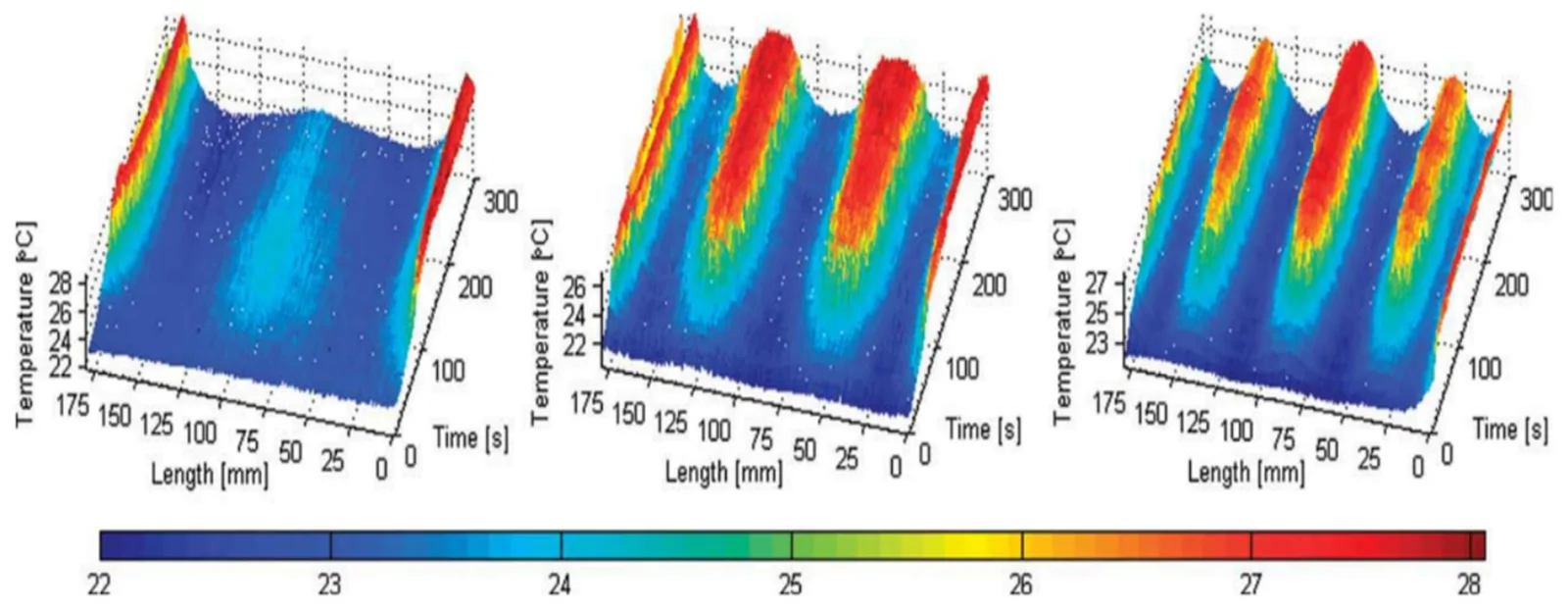
Time evolution of the temperature profiles for the first, second, and third resonant frequencies, from [114].

To get heat distribution over the whole geometry, it is important to know the thermal behavior of the polymer and understand the viscoelastic properties. Each vibration at different mode shapes and frequencies causes stress and strain in different regions of the PMC, so that, depending on viscoelastic characteristics, it is converted to heat as shown in Figure 10.

### 4.4. SHVT Performance, Detection Capabilities and Challenges

SHVT detects BVID with high sensitivity because a temperature increase of only a few degrees is sufficient to reveal differences and detect structural damage that is possible to achieve by this technique. Its performance has been demonstrated for various damage types, including simulated FBDs and BVID and low-velocity impact damage. Practical application has shown the detection of three damages from 20 J impacts with significantly elevated temperatures above background [80,82].

In the tests described in [95], the tested coupons of 250 × 10 × 2.5 mm (length × width × thickness) were excited multi-harmonically at three resonances between approximately 110 and 1076 Hz, with millimeter scale FBDs of four shapes; Amraei et al. [96] tested plates of 240 × 240 mm with thicknesses of 0.5, 0.75 and 1 mm, excited at the first natural frequency alone, between 44 and 85 Hz, with a single centrally located 50 mm square FBD. The shape dependence reported by Katunin and Wachla [95], in which notch-type defects were detected at milled depths where circular and rectangular defects were not, indicates that the stress concentration caused by the shape of the defect is a further variable that neither dataset resolves.

These two studies [95,96] that establish the depth sensitivity of SHVT show a consistent trend: detectability improves as the damage extends deeper into the specimen, i.e., as the material remaining above it becomes thinner. Amraei et al. [96] identified the damage in the raw thermograms in eight of nine scenarios spanning three plate thicknesses, the only exception being the thinnest plate (0.5 mm) with the shallowest FBD (0.05 mm). Katunin and Wachla [95] detected FBDs in 2.5 mm specimens once the remaining thickness fell to 1.75 mm, that is, 70% of the specimen thickness, and reliably from 1.5 mm, that is, 60%. These two results must not be combined into a single calibrated window. They were obtained on specimens whose thicknesses do not overlap, 0.5 to 1 mm in [96] against 2.5 mm in [95], so no interpolation between them is supported by measurement. The excitation differs as well: the coupons in [95] were driven multi-harmonically at three resonances between approximately 110 and 1076 Hz, the plates in [96] at the first bending mode alone, between 44 and 85 Hz. The dissipated power scales with the excitation frequency and with the square of the local strain amplitude, and the loss modulus is frequency dependent, so a given damage does not generate the same heat in the two configurations. The boundary conditions also impose different mode shapes. The two numbers are therefore reported as independent estimates: one bounds the smallest milled depth that produced a detectable signature in thin plates, the other the remaining thickness through which the signature could still be resolved in thicker coupons.

Thus, the two datasets indicate that the sensitivity of SHVT is governed by two independent factors acting on different length scales. The first is mechanical: the damage must be severe enough to cause the local stress distribution, since by Equation (22) the heat generated scales with the square of the local strain amplitude and with the volume over which that strain is elevated. The second factor is thermal: the heat generated at the damage must diffuse to the observed surface before the camera can register it. In the 2.5 mm coupons of [95] the signature became indistinct beyond a remaining thickness of approximately 1.75 mm. Whether that figure transfers to other thicknesses, lay-ups or excitation protocols has not been tested.

**Figure 11:**
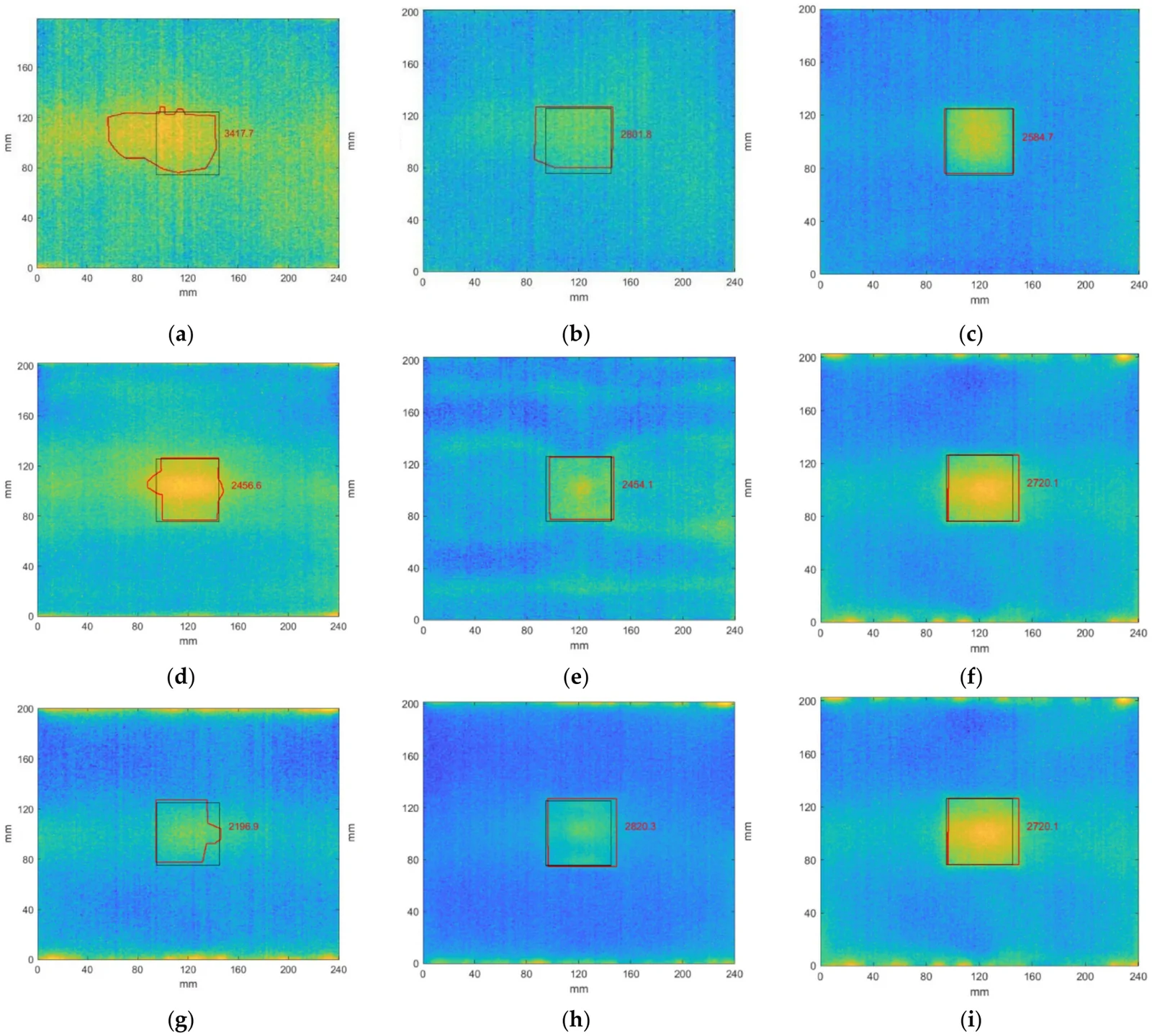
Enhanced thermograms of the nine SHVT scenarios with the quantified damage regions, for plates of thickness (**a**–**c**) 0.5 mm, (**d**–**f**) 0.75 mm and (**g**–**i**) 1 mm, at FBD depths of 10%, 25% and 50% of the thickness respectively in each row from left to right. The red contour is the identified damage boundary, the black contour the true one, and the annotated value the identified area in mm^2^. Reproduced from [96].

Beyond detection, SHVT also enables the extent of the damage to be quantified. Quantifying that agreement requires an explicit error criterion, since a comparison of identified and true areas alone can be satisfied by a contour of the right size in the wrong place. The hit/miss metric adopted in [96] and applied in [97] reports the proportion of the true damage area that the identified contour covers, and the proportion of the union of the two areas lying outside the true damage. For the GFRP plates processed in [97] the hit values were 92.80%, 94.10% and 98.06% for thickness reductions of 10%, 25% and 50%, with corresponding miss values of 18.98%, 12.52% and 21.61% (Figure 11). Coverage improves monotonically with the depth of the milled region; the overestimation does not, being largest for the deepest damage. Sizing accuracy and detection sensitivity are therefore not the same capability, and both components of the metric should be quoted.

Across all nine scenarios, the surface temperature rise remained below 2 °C. This bounds the thermal part of the question: the excitation leaves the specimen far below any temperature at which matrix properties change, unlike optical excitation, where the irradiated surface is the hottest point [96]. A low surface temperature rise is not, however, a demonstration on its own that the inspection is non-destructive. SHVT reaches that temperature by driving the structure at resonance, so the specimen accumulates mechanical load cycles throughout the test, and it is the cyclic strain rather than the temperature that carries the risk. For the exposures reported here, of the order of minutes at strain amplitudes well below the fatigue limit, no change attributable to the inspection was observed, and the criticality analyses in [13] indicate that degradation sets in only once the self-heating temperature approaches the range in which the storage modulus falls. Moreover, the experimental studies summarized in [13], which cover mechanical, thermomechanical, mechanochemical and microstructural analyses, confirm the non-destructive character of the applied loading and of its duration, including under ultrasonic-frequency loading [115].

**Figure 12:**
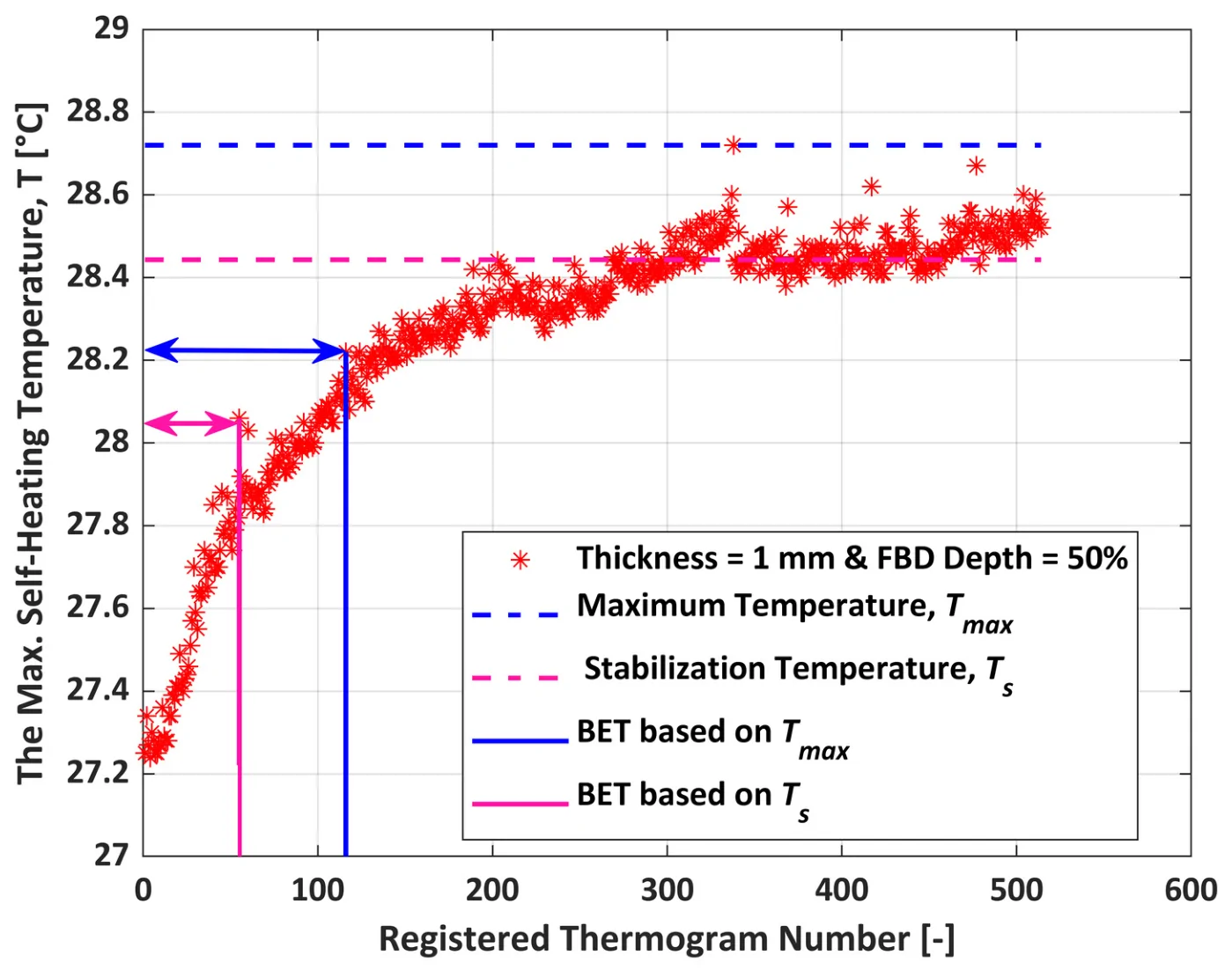
Determination of the BET from the evolution of the maximum self-heating temperature over the registered sequence, for a 1 mm plate with a 50% FBD. The dashed lines mark the maximum and the stabilized self-heating temperature; each BET is the frame index at which 1/e of the corresponding rise is reached. Reproduced from [96].

A practical difficulty specific to internally excited thermography is that the useful information is concentrated in a short window at the start of excitation, while a single SHVT test may register several hundred to several thousand thermograms. Because the self-heating temperature rises asymptotically towards a stabilized value, frames acquired late in the test carry the smallest spatial gradient and therefore the weakest damage signature, whereas the earliest frames are degraded by the non-uniform initial temperature distribution and by stabilization noise. Amraei et al. [96] addressed this with a two-step selection rule. The first step fixes an upper bound on the usable sequence, termed the boundary of effective thermograms (BET), by applying the thermal time-constant criterion to the recorded temperature history: the frame index at which the temperature has covered 1/e, that is approximately 63.2%, of its total rise towards either the maximum or the stabilized value. The two criteria give different bounds: 116 and 55 frames respectively for a 1 mm plate with a 50% FBD (Figure 12). The maximum self-heating temperature is reached later than the stabilized value and exceeds it, so 63.2% of the rise towards the maximum falls on a later frame. The maximum-temperature criterion is therefore the more permissive and the stabilized-value criterion the more conservative. The stabilized-value definition is used consistently in this review whenever a BET is quoted, and both frame counts are reported so that they can be compared on the same basis. The second step selects, within that window, the single frame that maximizes the ratio between the temperature rise averaged over the damaged region and the temperature rise of the damaged region relative to the plate as a whole. Applied to the nine scenarios, this reduced several hundred candidate frames to one per test without manual inspection, and the frames so selected lie at the beginning of the record, where the thermal gradient is steepest and the damage edges sharpest. The rule is effective but not unconditional, and three limitations should be recognised. First, it presumes a monotonic approach to a stable plateau; where ambient drift, intermittent contact at the coupling point or a shifting resonance makes the history non-monotonic, the 63.2% criterion can select a frame on a transient rather than on the true rise. Second, the selection follows the global temperature history rather than local contrast, so a frame optimal for the hottest region need not be optimal for a weaker second damage elsewhere. Third, the second step requires the damaged region to be identified in advance, so the criterion is a quantification aid applied after detection rather than a detection method in itself. Extending it to blind inspection remains open.

According to the research on vibrothermography reported in [116], the specimen vibrates at its natural resonance frequencies, and the component deformation profile takes the shape of the corresponding resonant mode. For efficient damage detection, the vibration amplitude near the damaged region should be non-zero. Hence, for a given resonant mode shape, damage located where the local strain amplitude is low produces little self-heating and may escape detection, which is why several modes are excited in turn. It is possible to measure the resonant mode shape and, in turn, the vibration coverage by applying a layer of viscoelastic polymer adhesive to the specimen surface before excitation [117]. Due to the dynamic strain continuity between substrate and adhesive, the polymer material dissipates heat according to the resonant mode shape of the underlying substrate. The heat intensity dissipated by the adhesive depends on the loss modulus of the material, along with the dynamic strain and vibration frequency. Therefore, by calculating the heat intensity and dynamic strain from the measured surface temperature and vibration respectively, the loss modulus of polymer adhesive can be estimated.

The amount of heat generated by the crack depends on the dynamic vibrational strain and excitation frequency [118], and therefore, a rapid and straightforward method for measuring dynamic strain is important. Because the specimen vibrates predominantly in resonant modes, the vibration pattern is nonuniform and results from a combination of the excited modes, producing regions of high and low vibrational amplitudes. Consequently, to predict the detectability of a crack at a specific location under vibration, it is necessary to determine the vibration pattern of a MUT. Existing methods for full-field dynamic vibration measurement are either excessively time-consuming or impractical for vibrothermographic applications [117].

It is useful to clarify the relationship between LDR (Section 4.2) and SHVT (Section 4.3), since the two are often discussed but address different aspects of the inspection process. LDR is fundamentally an excitation strategy: it identifies and exploits a frequency at which the defect itself resonates, thereby concentrating vibrational energy locally regardless of the underlying heat-generation mechanism [15,16,17,18,19,46]. SHVT, by contrast, is a heat-generation mechanism specific to viscoelastic polymer matrices, in which cyclic strain is converted into heat through hysteretic damping [11,94,114,119]. The two are frequently combined in practice: exciting a polymer composite structure at its LDR frequency increases the local strain amplitude at the damage, which, through Equation (22), directly increases the hysteretic heat generation rate, so LDR-tuned excitation can substantially enhance the sensitivity of SHVT-based inspection [93]. In terms of suitability, SHVT is inherently better matched to polymers and PMCs, since it relies on a viscoelastic loss mechanism that is largely absent in metals, whereas LDR is a more general excitation principle applicable to any material system where a defect produces a measurable local resonance, including metals inspected by friction-dominated ultrasonic vibrothermography (Section 4.1). Regarding flexibility, LDR is the more broadly applicable concept, as it can be paired with different heat-generation mechanisms and sensing modalities beyond thermal imaging, such as laser Doppler vibrometry. Regarding simplicity and inspection speed, SHVT alone, once an effective excitation frequency is known, offers a comparatively simple, single-step acquisition; the practical bottleneck for a combined LDR and SHVT inspection is the need to first determine the LDR frequency, which typically requires either a preliminary vibrometry scan or a broadband frequency sweep, adding time and instrumentation compared with fixed-frequency SHVT alone [15,16].

### 4.5. The Limitations of Mechanical Excitation

The preceding sections set out what mechanical excitation makes possible. The constraints are otherwise distributed across Section 4.1, Section 4.2, Section 4.3 and Section 4.4, so they are gathered here, and the conditions under which mechanical excitation is not the appropriate choice are stated explicitly.

The first group of constraints concerns the damage itself. Ultrasonic vibrothermography generates heat by friction and clapping at contacting faces, so it is effective for closed cracks, delaminations and disbonds that can rub under a load, and weak where the faces do not touch. A delamination held open by residual stress, a resin-rich void, distributed porosity or a bonded inclusion presents no sliding interface and produces little heat. Regions under high closure stress are also poor sources. SHVT reverses this dependence: its heat comes from viscoelastic hysteresis in the matrix, so contacting faces are not required, but the damage must perturb the local strain field enough to raise the dissipation above the background self-heating of the sound material, the condition quantified in Section 4.4. Damage that is compliant, small relative to the strain gradient, or where the matrix contributes little to the stiffness is correspondingly difficult to reveal.

The second group concerns the excitation and the specimen as a dynamic system. LDR and SHVT both depend on resonance, and therefore on knowing which frequency to apply. LDR requires the defect resonance, normally located by an auxiliary laser Doppler vibrometer scan or a frequency sweep, which adds an instrument the optical modalities do not need. SHVT requires the structural resonances from a modal analysis of the component. In both cases the frequencies shift with boundary conditions, temperature and the damage itself, so a protocol established on a coupon does not transfer unchanged to a mounted component. Damage lying in a region of low strain for the excited mode generates little heat and can be missed, which is why several modes are excited in turn. Anisotropy and lay-up also affect the mode shapes.

The third group is practical. Mechanical excitation must be introduced into the structure, which requires a shaker, a sonotrode or a bonded transducer, a coupling medium, and a fixture that holds the specimen repeatably. The result is a contact inspection, sensitive to the coupling pressure and to the condition of the coupling pad, with a risk of local damage at the coupling point in ultrasonic configurations. The fixture is not a detail: the mode shapes follow from the boundary conditions, so a change of clamping changes the detectability map. Large or geometrically complex components are difficult to excite uniformly, and the method does not scale to area as flash or scanned line heating does. Quantification remains the least mature aspect, since no depth-inversion relation of the maturity of Equation (5) exists for vibrothermography, so depth and severity are presently inferred rather than measured.

Overall together, mechanical excitation is the appropriate choice for closed, friction-active damage; for matrix-dominated damage in viscoelastic laminates; for inspection from one side only; for components where the admissible surface temperature rise is low; and where the damage is not amenable to purely diffusive detection. It is not appropriate for open or non-contacting defects, for porosity and other distributed features, for large-area screening under time constraints, for components that cannot be fixtured repeatably, or where an absolute depth measurement is required. For these the optical modalities of Section 3 remain the reference, and the two families are complementary rather than competing.

## 5. Signal Processing Techniques

Regardless of the type of excitation, the infrared camera produces the same kind of data: a sequence of thermograms in which every pixel has its own temperature time history. In PMCs, the trace that subsurface damage leaves in these histories is usually weak. It is also blurred by lateral heat diffusion and distorted by non-uniform heating, emissivity variation, environmental reflections, and detector noise [47,71,110,120,121,122]. Signal processing is used to deal with these problems. It enables extraction of information that is not visible in the time or frequency domain alone. In other words, it makes the damage more visible, removes artifacts, compresses a long sequence into a few informative images, and supports quantitative tasks such as estimating the depth and size of the damage [70,123,124].

The methods are reviewed in three groups: classical sequence-based transforms (Section 5.1), procedures for background suppression and quantitative characterization (Section 5.2), and machine learning-based methods (Section 5.3). One question is followed throughout: does a method rely on a model of heat diffusing from an external source? The answer decides whether the method can also be used for the internally heated sequences of vibrothermography described in Section 4.

### 5.1. Classical Sequence-Based Transforms and Derived-Image Processing

Most classical methods belong to one of four families [70]. Thermal contrast methods compare the temperature history of a suspect region with that of a sound (undamaged) region, following the definition of thermal contrast in Section 2.2. Thermographic signal reconstruction (TSR) fits a smooth curve to each history and then works with its derivatives. Fourier-based methods, with PPT (Section 3.4) as the main example, turn each history into amplitude and phase images. Principal component thermography (PCT), based on principal component analysis (PCA), reduces the whole sequence to a few dominant patterns. An important difference between the methods is how much they assume about heat transport. Some are built on the one-dimensional diffusion of heat from a surface pulse, while others work directly on the recorded data without any heat-transfer model.

The simplest methods look at each pixel separately. Differential absolute contrast (DAC) is a form of thermal contrast that does not need a sound reference region to be chosen by hand, because it estimates how a sound area would cool from the early part of the cooling curve [70]. Statistical features, such as the mean absolute deviation, central moments, skewness, and kurtosis, describe the shape of each pixel’s curve with a few numbers and flag pixels that behave differently from the background. They are quick to compute and often improve contrast straight away, but they become less reliable when the signal changes strongly over time or when the heating is non-uniform [125,126]. Similarity (correlation) maps compare the curve of every pixel with a reference curve. This helps to find small and shallow damage, but the result depends on the chosen reference and requires data without slow overall temperature drift [80,111]. Derivative operators, such as the gradient, the Laplacian, and the Hessian, respond to edges and sudden temperature changes. They sharpen damage boundaries that lateral diffusion has blurred, but they also amplify noise [111].

PCT looks at the sequence as a whole rather than pixel by pixel. Each thermogram is rearranged into a single line of numbers; these lines are stacked into a matrix, and singular value decomposition (SVD) splits the matrix into a set of patterns. The leading patterns, called empirical orthogonal functions, can be shown as images in which the damage-related contrast is concentrated, and the singular values rank the patterns by how much of the variation in the data they explain [70,80,127,128]. Because the patterns come from the data rather than from a fixed formula, PCT adapts to each specimen. In practice, the first two or three components carry most of the useful contrast. The first component, however, often shows the non-uniform heating pattern rather than the damage, so the components have to be selected with care [129].

Standard PCT assumes that the noise is small and roughly Gaussian. This assumption breaks down under strongly non-uniform heating, surface reflections, or outlier pixels, and two extensions deal with it. Robust PCT replaces the SVD with a low-rank plus sparse decomposition. In simple terms, the smooth, dominant part of the data is kept in one term, while isolated outliers and noise are moved to a second term so that they do not spoil the main components. On a CFRP plate containing FBDs, Teflon inserts, and pull-out defects, robust PCT raised the contrast-to-noise ratio (CNR), which measures how clearly a defect stands out from the background noise, by 40% on average compared with PCT. The gain was larger for flat-bottom holes (about 60%) than for pull-out defects (about 27%), but the computation took about 11.5 times longer [129]. Sparse PCT adds an L1 penalty, which forces many of the component weights to zero. Each component image then depends on fewer inputs, which makes it easier to interpret and helps to detect small, shallow damage [130].

Other studies reconstruct the sequence before decomposing it. Tang et al. [131] proposed a Markov-PCA method, in which the sequence is first reconstructed and then reduced by PCA. It improved the signal-to-noise ratio (SNR) of the feature image compared with PPT, although the result depended strongly on the starting frame and the length of the analysis window. Fleuret et al. [132] proposed a reconstruction based on linear support vector regression, which gave a higher CNR for small defects in CFRP and outperformed PCT and TSR-PCT at depths of 0.6 mm and 0.8 mm, but was still sensitive to non-uniform heating.

TSR, in contrast, is built directly on the diffusion model. According to Equation (7), the surface of a thick sample cools after a short pulse in a very predictable way: plotted on log–log axes, the cooling curve is a straight line with a slope of −1/2. TSR therefore fits a low-order polynomial, usually of fourth or fifth degree, to the logarithm of each pixel’s history, and the whole sequence is replaced by a few coefficient images [133]. The first and second derivatives of the curve are then calculated directly from these coefficients. Wherever damage disturbs the cooling, the curve departs from the straight line, and the derivative images show this clearly. They give much higher damage to background contrast than the raw thermograms and are largely unaffected by non-uniform illumination, emissivity variation, and spurious environmental radiation [133,134]. The second derivative suited defects shallower than 1 mm, while the first derivative was more accurate for deeper ones, which is a useful qualifier on the depth estimate from the derivative peak [135]. The time at which the second derivative peaks also provides the characteristic time used to estimate depth in Equation (5). Because TSR also reduces noise and compresses the data, it is most useful as a preprocessing and sizing step that improves the methods applied after it, rather than as a detector on its own. The results’ dependency on sound area location is a well-known problem of thermal contrast methods [56].

Phase-domain processing applies the pixel-wise DFT described in Section 3.4 to the temperature history [80,136,137]. Put simply, the phase describes when the thermal response arrives rather than how strong it is, which is why phase images are much less affected by non-uniform heating and emissivity. The phase also gives a depth estimate through the blind frequency and Equation (6) [138]. The practical value of this can be seen in aerospace carbon-fiber composites inspected by PT and line scan thermography [123]. Most of the artificial defects were detected by line scan thermography without advanced processing, at a CNR of at least 2.5, but the deepest defect, at about 3 mm, was detected only after signal processing and spatial filtering, which raised the CNR to 3.6.

**Figure 13:**
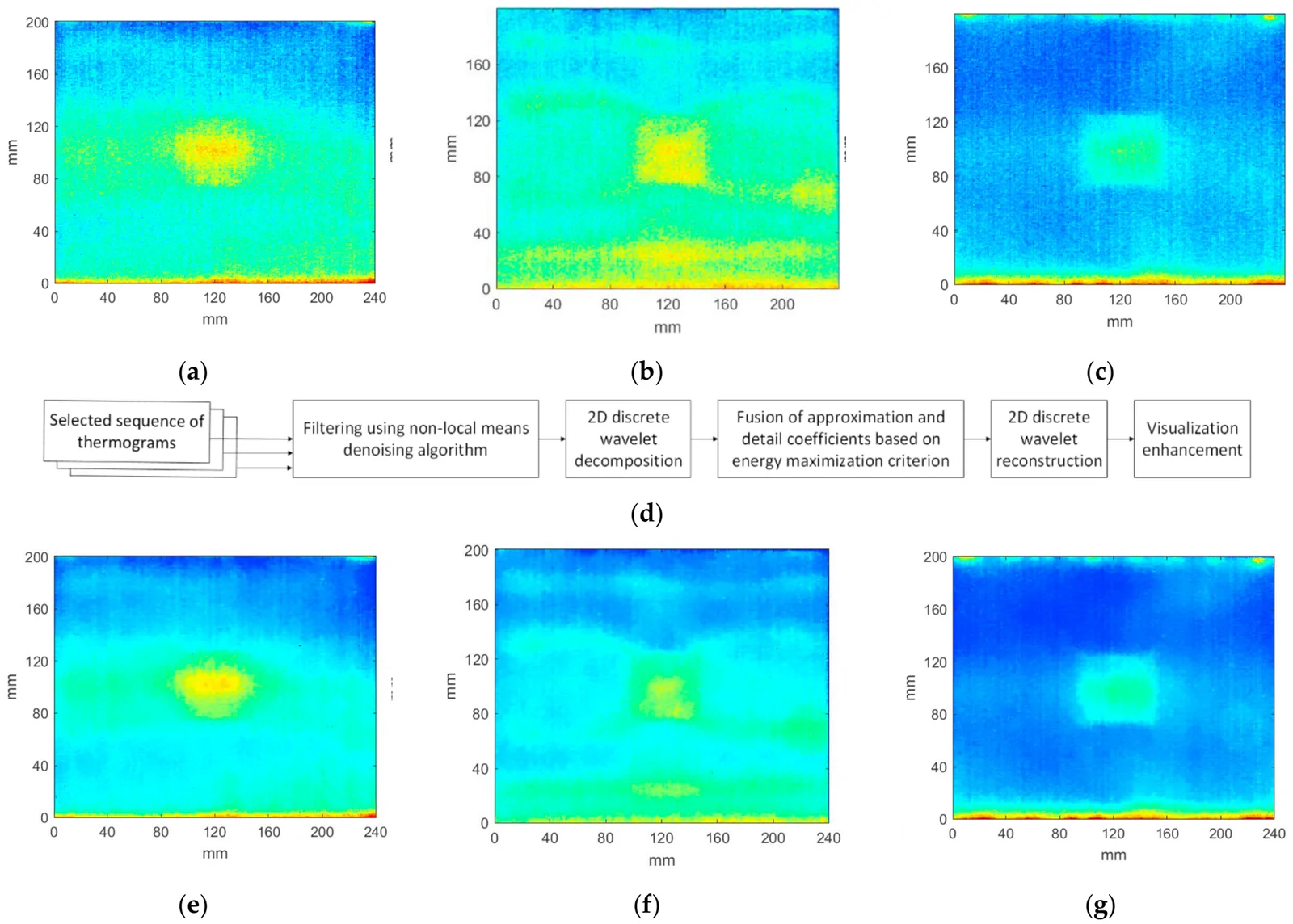
Enhancement of thermograms acquired by self-heating based vibrothermography in GFRP plates with 10%, 25% and 50% thickness reduction in the damaged area: (**a**–**c**) the best raw thermograms selected from the sequence; (**d**) the enhancement algorithm; (**e**–**g**) the corresponding fused enhanced thermograms. Adapted from [97].

Wavelet-based transforms split a signal, in time or in space, into different levels of detail or resolution. Coarse-scale (approximation) coefficients capture broad, slowly varying features such as the heating pattern, while fine-scale (detail) coefficients pick up the sharp local changes caused by damage. Keeping the detailed information while suppressing the coarse background therefore makes the damage stand out. How well this works depends strongly on the choice of mother wavelet and on the number of decomposition levels [80,139,140]. In practice, wavelets are mostly used as a denoising step inside the multi-stage pipelines of Section 5.2. The wavelet transform is particularly effective for detecting transient and localized damage due to its multi-resolution capabilities. Wavelet decomposition also serves as a fusion operator rather than a filter. In SHVT inspection of GFRP plates, five consecutive thermograms were denoised by non-local means filtering and fused through a single-level two-dimensional discrete wavelet transform under an energy-maximization criterion, using the biorthogonal 3.5 wavelet, after which Niblack thresholding delineated the damage [97]. Figure 13 shows the effect: boundaries distinguishable in the raw thermogram only for the deepest damage become bounded in all three cases after fusion, with the identified area covering 92.8% to 98.1% of the true damage.

Comparative studies show that no single transform is the best. In CFRP and GFRP laminates, LPT sequences processed by absolute thermal contrast, TSR, phase Fourier analysis, and PCA were compared with PT and ST. PCA applied to LPT gave the most accurate images, and the authors presented LPT with suitable processing as a cost-effective option for composites with low thermal conductivity and slow thermal response [141]. When TSR, PPT, and PCT were applied to the same pulsed dataset, TSR found the most defects, PPT gave the highest SNR for the deeper defects, and PCT gave the highest SNR for the shallower ones [142]. That study used steel rather than composites, but it shows that the best transform can change with defect depth.

### 5.2. Preprocessing, Background Suppression and Quantitative Characterization

A large part of the processing effort in composites is aimed at the background rather than at the damage itself. Finding a truly sound reference region is often difficult, so Erazo-Aux et al. proposed a procedure that works without one [120]. It combines a correction for non-uniform heating with oriented-gradient information, which describes the direction of local temperature changes, and with local and global segmentation. On CFRP specimens with Teflon inserts, it remained reliable under low-contrast, non-uniformly heated conditions without further preprocessing, and on that sample, it performed better than both a deep-learning method and a filtering-based background compensation [120].

A structured background can also come from the material itself. In woven CFRP, the weave creates a regular in-plane pattern that makes damage harder to detect and characterize, and PPT, PCT, or TSR applied alone do not remove it well [121]. Because the pattern repeats regularly, spatial Fourier filtering can separate the weave from the damage-related signal. This improves the spatial identification of delamination, although it does not give its thickness or depth [121]. When low contrast, noise, and non-uniform heating occur, multi-stage filtering works best. For CFRP phase images obtained by PPT, a framework that combined edge-preserving decomposition, adaptive detail enhancement, non-local means filtering, and wavelet denoising gave a higher CNR and clearer defect images than conventional PPT and PCT [143].

Model-based and decomposition-based workflows go one step further, from making damage visible to measuring it. In carbon/epoxy plates inspected by SPT, SVD was used to separate sound from defective zones. A thermal-quadrupole inversion, in which an analytical heat-transfer model of the plate is fitted to the measured data, then estimated both the depth and the thermal resistance of the defects, without prior knowledge of the material properties or the defect positions [124]. The heating and analysis durations influenced the results, which shows that such estimates depend on the chosen time window as much as on the model [124]. In the aerospace study mentioned in Section 5.1, PT was first used to measure the thermal diffusivity, and PPT was then used to estimate defect depth [123]. For deep defects in LPT data, Wu et al. [144] noted that existing methods suffer from limited feature extraction, high computational cost, manual intervention, and insufficient noise suppression. They proposed a dynamic compression reconstruction of the thermal signal to improve both the visualization of deep damage and the estimation of its depth.

### 5.3. Machine Learning and Deep Learning Methods

Machine learning-based methods shift thermographic processing from improving contrast toward automated interpretation that does not depend on the operator. The idea is not new: an early work [145] already used neural networks to classify pixels in processed contrast images and even to estimate depth classes. What has changed is the scope. Recent reviews describe learning models at several stages of the processing chain, including denoising, background removal, feature extraction, segmentation, and depth prediction [12,120]. Convolutional neural networks (CNNs), such as ResNet, U-Net, VGGNet, YOLO, and physics-guided networks, are used for these tasks and for detecting subtle signatures such as BVID [8]. Whatever the network, every implementation needs three things: a dataset, a set of features, and an algorithm that separates sound from damaged regions [146].

It helps to know what these methods produce. Classification gives a label to an image or a region, for example, damaged or sound, or a type of damage. Detection draws a box around each defect, which is often enough for screening. Segmentation labels every pixel, which is needed when the size and shape of the damage have to be measured. Most methods are supervised, meaning that they learn from examples labeled by an expert.

**Figure 14:**
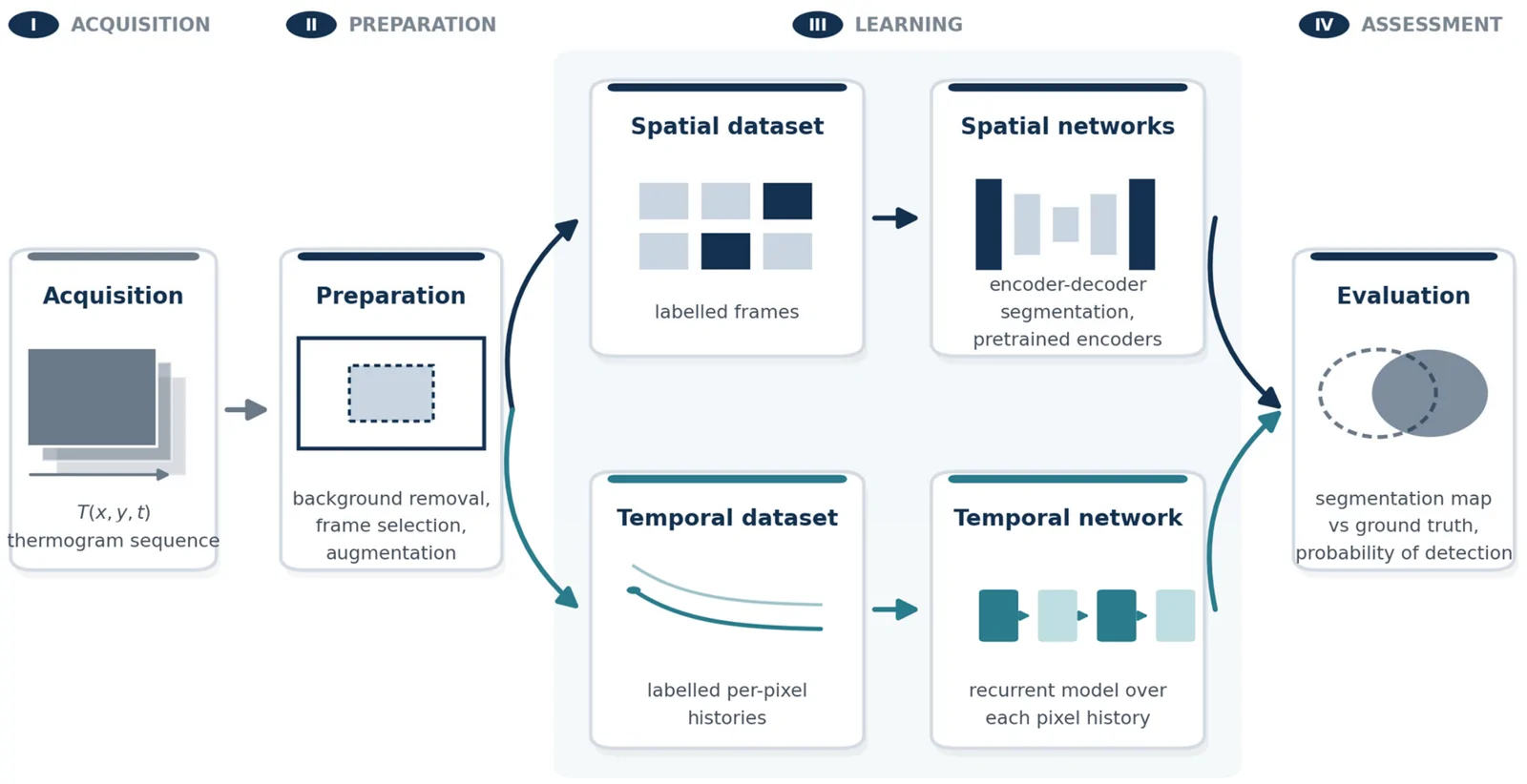
Deep-learning workflow for defect segmentation in thermographic sequences. Drawn after [150]. Diagram drawn with the assistance of Claude (Anthropic, claude.ai); the content was specified and verified by the authors.

Segmentation and localization form the largest group of studies, because they reduce the dependence on the operator while keeping pixel-level information about the damage [70,120,147]. In polymer components containing FBDs of different sizes and depths, Mosavifard and Farahani [148] combined several classical representations (PCA, independent component analysis, TSR, partial least squares regression, and contrast-limited adaptive histogram equalization) with a U-Net network built on a MobileNetV2 backbone. They obtained 96.55% accuracy, 85.96% recall, 93.84% precision, a Dice coefficient of 89.38%, and an intersection over union (IoU) of 80.97%. Dice and IoU both measure how well the predicted defect area overlaps the real one, with 100% being a perfect match. The best combination, PCA followed by U-Net, found all 25 defects, which corresponds to a probability of detection (POD) of 100% [148]. The same study summarized earlier work in which networks such as Mask R-CNN, YOLO, U-Net, and DeepLabV3+, applied to PT and ST images, reached, in the best cases, an IoU above 90% and a POD of about 0.90–0.92 [148]. On CFRP data, spatial networks such as U-Net and DeepLabV3, combined with recurrent networks that follow the signal over time, have also been compared with simpler adaptive-thresholding methods [149]. A hybrid architecture of this kind separates the recorded sequence into a spatial dataset of labelled frames and a temporal dataset of per-pixel histories, trains a segmentation network on the first and a recurrent network on the second, and judges both against the same ground truth by the probability of detection (Figure 14) [150].

Classification of the damage state forms the second group. In the simplest implementations, the classifier does not work on the images themselves but on a few numbers extracted from them. Alhammad et al. [146] acquired PT sequences of impacted CFRP panels, processed them with PPT and PCT, extracted statistical descriptors, and classified them with a cubic support vector machine (SVM), a classifier that finds the boundary that best separates two classes. Across ten panels of 1.6 mm and 3.8 mm thickness impacted at 4–12 J, the accuracy ranged from 78.7% to 93.5%, and reflection mode was the more reliable acquisition mode because the damage lay close to the heated surface [146]. Moskovchenko and Svantner [151] compared several processing methods and classifiers for flash PT of impact-damaged CFRP. The highest balanced accuracy, 96.7%, came from *k*-nearest neighbors, which labels a sample according to the most similar examples in the training set, applied to DAC data, followed by a Gaussian support vector machine on raw data (95.1%), k-nearest neighbors on TSR derivative data (94.9%), and a neural network on statistical features (94.3%) [151]. The processing method and the classifier therefore have to be chosen. Deep networks can also classify the type of damage directly: dense convolutional networks trained on five classes (debonding, water ingress, delamination, cracking, and sound material) achieved high accuracy on CFRP laminates and honeycomb sandwich specimens [152].

A third group needs no labeled data at all. These unsupervised methods learn the normal behavior of the material and flag departures from it. An autoencoder is a network that learns to compress its input and then rebuild it. In deep autoencoder thermography, the network is trained on damage-free thermal behavior, and the activation of its hidden layers are shown as images. This extracts nonlinear damage features, and the method outperformed PCT-based methods on carbon-fiber specimens [153]. One-dimensional convolutional versions work on the temporal signal of each pixel rather than on whole images [154]. In this sense, an autoencoder does the same job as the data-derived patterns of PCT, but with a nonlinear model instead of a linear one.

Automation does not always require deep learning. Gómez and Castro [155] applied PPT and PCT to GFRP specimens and then used a simple SNR criterion to detect defects automatically, which worked for defects of 5 mm diameter at 3 mm depth. Automated interpretation therefore ranges from simple threshold rules to segmentation networks, rather than being split into classical and learning-based processing [70,155]. Hybrid systems combine both ends of this range. For wind-turbine blades, Bounenni et al. [147] combined infrared image comparison with U-Net segmentation, so that each pathway checked the other and false positives were reduced, while U-Net kept pixel-wise localization over a large structure.

Supervised learning needs many labeled examples, but real experimental data is scarce. Several studies therefore train on simulated thermograms. Fang et al. [156] trained Mask R-CNN on images from finite-element simulations combined with experimental ones and reported better segmentation on acrylic and CFRP specimens. Tong et al. [157] used a parameterized three-dimensional model to generate the training set for a Faster R-CNN framework, which was then tested on experimental coupons and on aerospace panels with production defects. Simulation lets a model see a wider range of defect depths and shapes during training, but testing on independent components and on damage that was not introduced on purpose is still necessary.

Physics can also be built into the network. Lim et al. [158] used a physics-informed neural network (PINN), whose training also had to satisfy the heat diffusion equation and its boundary conditions, to estimate the background temperature of pulsed CFRP thermograms. Subtracting this estimate made the defect-related differences easier to see. How far such a model can be trusted depends on how well its assumed boundary conditions describe the real specimen. For depth, Zheng et al. [159] took the logarithmic slopes of pulsed cooling curves, the same quantity that TSR uses, and encoded them as small two-dimensional images, so that a CNN could detect defects and estimate their depth in one step. This approach outperformed feed-forward, one-dimensional convolutional, and recurrent networks on FBD specimens, although the authors noted that other defect types would require further training.

These results should be read with care, for three reasons. First, machine learning-based processing is not always better. On the CFRP sample discussed in Section 5.2, the reference-free analytical method outperformed a deep-learning method under low-contrast, non-uniformly heated conditions [120]. In addition, classical processing is still part of most learning workflows, either to improve the input or as the representation on which the network is trained [146,148,151]. Second, performance depends on the test setup. It changes with specimen thickness, the position of the damage relative to the heated surface, and the acquisition mode [146], and a study of a temporal CNN for LT inspection of CFRP examined how its performance changed with the number of excitation cycles, the number of frames per cycle, and the share of randomly lost frames [160]. Third, published metrics such as accuracy, recall, IoU, Dice, POD, SNR, and CNR depend on the task and the dataset [146,148]. Reported accuracies range from about 80% for conventional CNN classifiers to 99% for newer architectures, but they refer to different tasks, damage types, and specimen sets, so they cannot be read as a ranking [8]. A fair comparison would need common benchmark datasets. The choice of processing method should be guided by nature of signal and specific NDT objective rather than by computational algorithm alone.

The main limitation is still the quality, quantity, and variety of the training data. PINNs, simulation-based training, and digital-twin model updating are the main responses being developed, and they point toward inspection that predicts how damage will develop rather than only diagnosing it [8]. The studies above that combine simulated and experimental data [156,157] or build diffusion physics into training [158] already show this direction. For the subject of this review, there is a more specific gap. Learning-based processing has developed mainly for optically excited thermography, particularly PT, while vibrothermography has not benefited to the same extent. PCA variants, advanced computational processing, and deep learning are still rarely used to quantify damage in vibrothermographic data, especially delamination and BVID [12]. The reasons for that imbalance are structural rather than incidental, and three can be identified. The first is the supply of labelled data: supervised segmentation networks are trained on hundreds to thousands of annotated frames, and the public pulsed-thermography datasets have no vibrothermographic equivalent. An SHVT campaign yields one informative frame per excitation once the selection rule of Section 4.4 has been applied, so a comparable corpus requires orders of magnitude more experiments. The second is that the forward problem is harder to simulate, which removes the usual remedy for scarce data: synthetic sets for pulsed thermography follow from the linear diffusion equation, whereas internal heat generation is a coupled thermo-mechanical problem whose source term depends on the local strain field, on the frequency- and temperature-dependent loss modulus, and on the damage geometry. The third is transferability: the heat source depends on the mode shape and the mode shape on the boundary conditions, so a model trained on one specimen and fixture does not generalize as a model trained on flash data transfers between flat panels. The three point to the same remedies: open benchmark datasets for vibrothermography, physics-based generators of synthetic sequences, and architectures that take the excitation parameters and the modal response as inputs.

The closest existing example involves heat produced by the structure itself. In wind-turbine blades under cyclic fatigue loading, the damaged regions heat up, and the AQUADA system used passive thermography and automated image processing to locate, segment, and measure the damage in one step, with a reported potential to halve the inspection cost [161]. Later versions replaced simple intensity thresholding with deep learning segmentation to cope with changing thermal backgrounds on operating turbines [162].

Table 3 collects the families reviewed in this section and compares them on the properties that decide their selection in practice: the data they take as input, the result they return, whether a reference measurement or a training set is required, their behavior for shallow and for deep damage, their sensitivity to non-uniform heating and to emissivity variation, their computational cost and their level of maturity. The entries follow the studies cited in the first column; where a qualitative judgement is given reflects the conditions reported in those studies rather than a common benchmark.

## 6. Conclusions

This review examined active IRT for PMCs from the standpoint of heat generation. Optical and vibration-based excitation are separated less by the cameras and processing chains they share than by where the heat originates, and that single distinction propagates into every practical aspect of an inspection: the attainable contrast, the depth reach, the admissible excitation power, and the confidence with which damage can be quantified and sized.

For optically excited modalities, the governing physics is linear diffusive transport from an external source, and it is well characterized. This is why PT and PPT support quantitative depth inversion through the time of peak contrast and the blind frequency, respectively, and LT through the thermal diffusion length. The corresponding limitation is equally structural: heat must travel from the surface to the damage and back before any contrast appears, so the contrast degrades rapidly with damage depth, and raw amplitude images remain vulnerable to emissivity variation and non-uniform heating. Phase-based processing mitigates the latter, but not the former.

Mechanical excitation removes that round trip by generating heat at the damage itself, and the three branches reviewed here do so by different means. Ultrasonic vibrothermography relies on friction and clapping at crack faces and is therefore most effective for closed cracks. LDR is not a heat-generation mechanism but an excitation strategy that concentrates vibrational energy at the damage; its cost is the need for prior knowledge of the defect resonance, usually obtained from an auxiliary vibrometry scan. SHVT exploits the viscoelastic hysteresis of the matrix itself, which makes it specific to polymers and polymer composites and allows large-area, low-power, single-sided inspection at a temperature rise of only a few degrees. LDR and SHVT are complementary rather than competing: LDR-tuned excitation raises the local strain amplitude, and the hysteretic heat generation scales with its square.

The quantitative maturity of the two families is not comparable. The two datasets that establish the depth sensitivity of SHVT point to two separate limits, each acting on a different quantity. The damage must produce a stress concentration strong enough to raise the local strain under resonant vibration; in the flat-bottom-damage specimens studied, this condition was met over a depth range beginning near 0.1 mm, with the shallowest case sized least reliably. The damage must also lie close enough to the observed surface for the generated heat to reach it, which was the case up to roughly 1.75 mm of remaining thickness. The first limit concerns the depth of the damage, the second the thickness of the material above it. The two limits were, however, established in experiments whose thickness ranges do not overlap, so their combination remains an interpretation rather than a directly tested result. SHVT not only detects damage but also enables its size to be estimated, although detection and sizing improve under different conditions. The damage was identified in eight of the nine scenarios reviewed, and the hit/miss metric gave coverage of the true damage between 92.8% and 98.1%, with 12.5% to 21.6% of the identified region lying outside it, the overestimation being largest for the deepest damage. The surface temperature rise of less than 2 °C bounds the thermal risk. No depth-inversion relation analogous to those of optical thermography has yet been validated for vibrothermography, and no general model links the stress concentration caused by the damage, the interface condition and the dissipated energy to the resulting surface thermal field.

On the processing side, no single family dominates. Classical sequence transforms such as PCA, PCT and TSR remain the established choice for enhancing the visibility of damage, and phase-domain processing reduces heating artefacts while preserving subsurface information. Procedures directed at the background, from the correction of non-uniform heating to the spatial filtering of the weave pattern, address the impairment that limits all of them, while model-based inversions extend processing from visibility enhancement to the quantification of depth and thermal resistance. Machine learning-based methods offer operator-independent interpretation, but they remain dependent on training data that the field does not yet possess in sufficient quantity or diversity, and they do not uniformly outperform well-designed analytical processing. Reported metrics are task- and dataset-specific and should not be compared across studies. A further asymmetry is that machine learning-based processing has developed mainly within optically excited thermography, whereas its integration with vibrothermography, in particular for the quantification of delamination and BVID, remains largely unexplored.

Three priorities follow from this synthesis. First, an experimentally validated relationship linking the stress concentration caused by the damage, the dissipated mechanical energy, and the surface temperature is required before vibrothermography can deliver sizing of the kind routinely obtained from ultrasonics. Second, standardized reference specimens are needed so that the sensitivity of different techniques can be compared directly, with detection limits stated in absolute terms with the specimen thickness. Third, physics-informed and simulation-augmented learning offers the most realistic route from laboratory demonstration to routine industrial inspection of PMCs, and extending it to vibrothermography, where machine learning-based processing is still little used, is a natural next step.

## Figures and Tables

**Table 1:** Comparison of NDT techniques with explicit indication of applicability to PMCs.

NDT Technique	Flaw Type	Sensitivity	Environmental Testing Possibility	Cost	Applicability to PMCs
Visual Testing	Surface cracks, visible delaminations, corrosion, visible impact damage, surface voids	From mm	Yes	Low	Yes, but screening only; insensitive to BVID and all internal damage
Penetrant Testing	Surface cracks, surface delaminations, visible impact damage, surface discontinuities, welding defects	From micrometer	Yes	Low	Limited; porous or textured composite surfaces retain penetrant and give false indications. Used mainly on metallic fittings
Magnetic Testing	Cracks, erosion and corrosion losses (metals)	From micrometers	Yes	Low to medium	Not applicable to PMCs; listed for reference only, as it is used on associated metallic fasteners and fittings
Ultrasonic Testing	Internal cracks, delaminations, impact damage, foreign object inclusions, porosity, internal discontinuities	From micrometers	Yes/No, depending on technique and coupling method	Medium–High	Yes; the established reference technique for depth sizing of delaminations
Radiographic Testing	Internal/external cracks, corrosion, delaminations, inclusions, voids, porosity	From micrometers	Limited/sometimes yes	High	Partial; insensitive to planar delaminations oriented perpendicular to the beam, which is the dominant impact-damage morphology
Eddy Current Testing	Surface and near-surface cracks, erosion, corrosion losses; useful for conductive materials and carbon fiber reinforced polymer (CFRP) in some cases	From micrometers to millimeters near surface	Yes	Medium	Partial; requires electrical conductivity, so applicable to CFRP but not to glass fiber reinforced polymer (GFRP)
IRT	Surface/subsurface cracks, delaminations, corrosion, disbonds, impact damage	Hundreds of micrometers	Yes	Medium–High	Yes; fast, full-field, single-sided screening. Depth quantification is less reliable than ultrasonics
X-ray CT	Internal cracks, voids, porosity, inclusions, delaminations, complex 3D defects	From nanometers in high-resolution systems	No	Very High	Yes; used as ground truth for validating faster field techniques
Optical Coherence Tomography	Surface and near-surface microcracks, cracks, delaminations, voids, pores	From micrometer	Yes	High	Limited; requires a translucent matrix, so applicable to some GFRP but not to CFRP.

**Table 3:** Processing techniques and their usefulness for specific operations in the thermographic inspection of PMCs.

Processing Technique	Operates On	Contrast Enhancement	Background and Noise Suppression	Localization and Sizing	Depth Quantification	Main Requirement or Limitation	Demonstrated Scope and Maturity
Thermal contrast, DAC [70]	raw thermograms relative to sound reference; DAC computes an ideal sound signal from early frames [163]	Moderate	Moderate: DAC removes the need to choose a sound area [163]	Moderate: in a classifier comparison the highest balanced accuracy, 96.7%, came from KNN applied to DAC data [151]	Low: modified DAC with thermal quadrupoles enables quantification [163]	one-dimensional semi-infinite assumption; accuracy falls as the heat front reaches the rear face [163]	Mature and widely used; demonstrated on embedded inserts in a Kevlar panel and on 10 mm flat-bottom holes at 1–3.5 mm depth [70]
TSR [133]	per-pixel cooling curve fitted by a log-log polynomial; first and second derivatives [164]	High: derivative images reduce blurring [164]	High: maximal SNR and least susceptible to uneven heating and lateral heat diffusion [135]	High: sharper defect edges [164]	Moderate: depth and thermal resistance from derivative times [164]	built on one-dimensional flash-heating theory; the polynomial degree must be optimized for each case [165]	Mature; implemented in commercial flash-thermography systems
Phase-domain processing (PPT) [80,136,137,138]	per-pixel Fourier transform; phase and amplitude spectra [80,136,137]	High	High: insensitive to non-uniform heating and emissivity [80,136,137]	Moderate	High: blind frequency [138]; robust for depth in CFRP [123], with the coefficient A equal to 1.73 [135]	trade-off between sampling rate, truncation window and computing time; the diffusivity is needed for depth [70]	Mature; the usual choice when the depth itself has to be retrieved
PCT (PCA, SVD) [70,80,127,128]	whole sequence as a matrix; orthogonal modes by SVD [70,80,127,128]	High: PCA on long-pulse data gave the most accurate imaging [141]	High: noise rejection and data compression [70,80,127,128]	High: PCA with U-Net found 25 of 25 defects [148]	Low: the characteristic time relates to depth [127]	sensitive to noise and outliers [129]	Mature; the usual baseline against which newer methods are compared
Robust and sparse PCT [129,130]	low-rank background plus sparse defect matrix, or sparse loadings [129,130]	High: CNR about 40% above PCT [129]	High: multi-scale wavelet transform with sparse PCT removes background and noise [166]	Moderate: Jaccard index 0.74 against 0.70 for PCT [129]	not addressed	11.5 times slower than PCT [129]	Emerging; the reported gain concerns low-contrast damage and comes from single datasets
Sequence reconstruction (Markov-PCA, SVR) [131,132]	reconstructed sequence then PCA [131]; per-pixel regression [132]	High: SNR gain above PPT [131]	High: removes noise and redundancy [131]	Moderate	Moderate: Markov-PCA with back-propagation predicted depth and diameter, error about 4 to 10% [167]	results depend on the start frame and window size [131]	Emerging; the result depends on the analysis window chosen
Wavelet decomposition [80,139,140]	temporal signals (one-dimensional DWT) or images (two-dimensional multi-scale) [80,139,140]	Moderate: raises PPT contrast with non-local means [143]. fusion of consecutive thermograms sharpens damage edges in SHVT [97]	High: multi-scale wavelet band-pass removes background and high-frequency noise [166]	High: wavelet with gradient filtering, 751 rivets scanned [168]; resolution enhancement [169] hit rate 92.8% to 98.1% for SHVT damage contours [97]		usually a preprocessing stage; the wavelet and the scale must be chosen the number of fused thermograms is set empirically [80,97,139,140]	Established as a preprocessing filter, mainly where a periodic background dominates
Background suppression (heating correction, spatial filtering) [120,121]	individual thermograms in the spatial domain [120]	Moderate	High: Gaussian background model beat DAC and filtering-based compensation [170]	High: with oriented gradients it beat a deep-learning model [120]	Moderate: estimated and actual depths compared [120]	an additional background compensation is needed for real slabs; the adjusted method cut residual non-uniformity by 72%, against 39% for normalized contrast and 25% for modified DAC [171]	Established for full-field correction; several competing formulations
Model-based inversion (SVD with thermal quadrupoles) [124]	sequence fitted to an analytical layered heat-transfer model [124]	Moderate	Moderate	Moderate	High in principle: fits the defect parameters [124]	thermal quadrupoles gave inferior depth estimates because of noisy raw signals, and non-linear fitting is time-consuming and hardly applicable to full-format images [135]	Moderate and computationally heavy; demonstrated on 2.35 mm carbon/epoxy plates with 10 mm inserts at several depths [124]
Classical classifiers (SVM, KNN) [146,151]	engineered features: statistics, TSR coefficients, DAC values [146]	not addressed; operates on processed input	depends on the input features	High: SVM accuracy 78.7 to 93.5% [146]; an unsupervised one-class SVM was reported as more accurate than the supervised SVM [172]	Moderate: RBF networks and SVM for depth quantification [145]; KNN depth classes at 98.8% [173]	performance hinges on preprocessing and feature choice [151]	Moderate; demonstrated on impact-damaged CFRP panels 0.7–3.8 mm thick [146,151], and dependent on the features chosen
Deep segmentation and detection (U-Net, Mask R-CNN, YOLO) [148,156]	single frames or processed images (PCA, TSR) [148]	indirect	Moderate: cross-network learning significantly minimizes uneven illumination [150]	High: Dice 89.4%, IoU 81.0% [148]; Mask R-CNN segmentation [156]	Low unless multi-task: relative depth error below 10% [174]	needs large labelled datasets; synthetic finite-element data helps [156]	Emerging; limited by the amount of labelled data
Unsupervised methods (autoencoders, low-rank) [153,154]	raw or processed sequences, without labels [153]	High: autoencoder hidden layers highlight defects [153]	High: denoising and background removal [175]	Moderate: k-means clustering [76]	Low: emerging [175]	the model depth and the number of clusters must be chosen [153]	Emerging; few independent validations
Physics-informed networks [158]	thermograms constrained by the heat equation [158]	High: through background removal [158]	High: reconstructs the non-uniform background [158]	Moderate: PINN predictions subtracted before PCT [176]	Moderate: depth inversion errors below 7% for shallow and moderately embedded defects in GFRP, rising to about 10% for the deepest [177]	needs governing equations and boundary conditions; few experimental validations [158]	Early; few experimental validations

Note: the qualitative judgements in this table summarize behaviour reported in the studies cited in the first column. Those studies used different specimens, excitation configurations and damage types, and no common benchmark dataset exists across these families, so the entries are not directly comparable and the techniques cannot be ranked from them. The scale and maturity column reports what has been demonstrated for polymer-matrix composites, not a bound on what is achievable. Reported metrics depend on the task and the dataset and should be compared only within a single study.

## Data Availability

No new data were created or analyzed in this study. Data sharing is not applicable to this article.

## Abbreviations

The following abbreviations are used in this manuscript:

AHT	abnormal heating thermography
BET	boundary of effective thermograms
BVID	barely visible impact damage
CFRP	carbon fibre-reinforced polymer
CNR	contrast-to-noise ratio
CT	computed tomography
DAC	differential absolute contrast
Dice	Dice similarity coefficient
DMA	dynamic mechanical analysis
FBD	flat-bottom damage
FMT	frequency modulated thermography
FRF	frequency response function
GFRP	glass fibre-reinforced polymer
IoU	intersection over union
IRT	infrared thermography
LDR	local defect resonance
LDV	laser Doppler vibrometer
LPT	long pulse thermography
LT	lock-in thermography
NDT	non-destructive testing
PCA	principal component analysis
PCT	principal component thermography
PINN	physics-informed neural network
PMC	polymer matrix composite
POD	probability of detection
PPT	pulsed phase thermography
PT	pulsed thermography
SHT	surface heating thermography
SHVT	self-heating based vibrothermography
SNR	signal-to-noise ratio
ST	step heating thermography
SVD	singular value decomposition
SVM	support vector machine
TSR	thermographic signal reconstruction
UT	ultrasonic vibrothermography
VHT	volume heating thermography
